# The Free Energy Landscape of Small Molecule Unbinding

**DOI:** 10.1371/journal.pcbi.1002002

**Published:** 2011-02-03

**Authors:** Danzhi Huang, Amedeo Caflisch

**Affiliations:** Department of Biochemistry, University of Zürich, Zürich, Switzerland; Max Planck Institute for Biophysical Chemistry, Germany

## Abstract

The spontaneous dissociation of six small ligands from the active site of FKBP (the FK506 binding protein) is investigated by explicit water molecular dynamics simulations and network analysis. The ligands have between four (dimethylsulphoxide) and eleven (5-diethylamino-2-pentanone) non-hydrogen atoms, and an affinity for FKBP ranging from 20 to 0.2 mM. The conformations of the FKBP/ligand complex saved along multiple trajectories (50 runs at 310 K for each ligand) are grouped according to a set of intermolecular distances into nodes of a network, and the direct transitions between them are the links. The network analysis reveals that the bound state consists of several subbasins, i.e., binding modes characterized by distinct intermolecular hydrogen bonds and hydrophobic contacts. The dissociation kinetics show a simple (i.e., single-exponential) time dependence because the unbinding barrier is much higher than the barriers between subbasins in the bound state. The unbinding transition state is made up of heterogeneous positions and orientations of the ligand in the FKBP active site, which correspond to multiple pathways of dissociation. For the six small ligands of FKBP, the weaker the binding affinity the closer to the bound state (along the intermolecular distance) are the transition state structures, which is a new manifestation of Hammond behavior. Experimental approaches to the study of fragment binding to proteins have limitations in temporal and spatial resolution. Our network analysis of the unbinding simulations of small inhibitors from an enzyme paints a clear picture of the free energy landscape (both thermodynamics and kinetics) of ligand unbinding.

## Author Summary

Most known drugs used to fight human diseases are small molecules that bind strongly to proteins, particularly to enzymes or receptors involved in essential biochemical or physiological processes. The binding process is very complex because of the many degrees of freedom and multiple interactions between pairs of atoms. Here we show that network analysis, a mathematical tool used to study a plethora of complex systems ranging from social interactions (e.g, friendship links in Facebook) to metabolic networks, provides a detailed description of the free energy landscape and pathways involved in the binding of small molecules to an enzyme. Using molecular dynamics simulations to sample the free energy landscape, we provide strong evidence at atomistic detail that small ligands can have multiple favorable positions and orientations in the active site. We also observe a broad heterogeneity of (un)binding pathways. Experimental approaches to the study of fragment binding to proteins have limitations in spatial and temporal resolution. Our network analysis of the molecular dynamics simulations does not suffer from these limitations. It provides a thorough description of the thermodynamics and kinetics of the binding process.

## Introduction

A wide variety of physiological processes and biochemical reactions are regulated by the binding of natural ligands to proteins. Furthermore, most known drugs are small molecules that, upon specific binding, modulate the activity of enzymes or receptors. Several experimental techniques for fragment-based drug design have been developed in the past 15 years and successful applications have been reported (see for a review [1], [2]). At the same time, a plethora of computer-based approaches to small-molecule docking have been developed and applied to a wide variety of protein targets. These *in silico* methods make use of simple and efficient scoring functions and are based mainly on stochastic algorithms, e.g., genetic algorithm optimization of the ligand in the (rigid) substrate-binding site of an enzyme [3], [4]. Only recently, explicit solvent molecular dynamics (MD) simulations have been used to investigate the binding of small fragments to proteins at atomistic level of detail, which is very helpful for the design of small-molecule inhibitors [5], [6], [7], [8]. Out of equilibrium simulations of pulling have been carried out for an hapten/antibody complex [9] and small molecule inhibitors/enzyme complexes [10], but it is not clear how much the external pulling force alters the free energy surface.

In the past five years, new methods based on complex networks have been proposed to analyze the free energy surface of folding [11], [12], [13], [14], [15], [16], [17], [18], [19], which governs the process by which globular proteins assume their well-defined three-dimensional structure. These methods have been used successfully to analyze MD simulations thereby revealing multiple pathways and unmasking the complexity of the folding free energy surface of 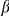-sheet [11], [13], [20], [21], [22] and 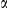-helical [23], [24], [25] peptides, as well as small and fast-folding proteins [26], [27], [28], [29]. Yet, no network analysis of the free energy surface of ligand (un)binding has been reported as of today. There are two main reasons for investigating the (un)binding free energy landscape. First, a wide variety of biochemical processes are regulated by the non-covalent binding of small molecules to enzymes, receptors, and transport proteins, and the binding/unbinding events are governed by the underlying free energy surface. Second, the characterization of metastable states within the bound state is expected to help in the identification of molecular fragments that bind to protein targets of pharmacological relevance, which could have a strong impact on experimental [2] and computational [4] approaches to fragment-based drug design.

Here we use complex network analysis [11] and the minimum cut-based free energy profile (cut-based FEP) method [13] to study the free energy landscape of the bound state and the unbinding pathways of six small ligands of FKBP sampled by explicit water MD at physiological temperature. These compounds were chosen not only because of the knowledge of their binding mode (X-ray structures of three of them) but also because their experimentally measured dissociation constants are in the mM range [30]. Therefore, we expected that several events of spontaneous ligand unbinding from FKBP could be sampled by running independent MD simulations starting from the bound state without any external bias and within a 20-ns simulation time (which requires about four days on a commodity processor).

## Materials and Methods

### MD simulations

The coordinates of FKBP in the complex with dimethylsulfoxide (DMSO), methyl sulphinyl-methyl sulphoxide (DSS), and 4-hydroxy-2-butanone (BUT) were downloaded from the PDB database (entries 1D7H, 1DHI, and 1D7J, respectively). The starting conformation of tetrahydrothiophene 1-oxide (THI), 5-hydroxy-2-pentanone (PENT), and 5-diethylamino-2-pentanone (DAP) were prepared manually by overlapping the (CH
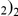
SO group of THI to the DMSO atoms in the DMSO/FKBP structure, while the (CH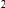)
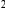
CO group of PENT and DAP was overlapped to the corresponding atoms in BUT. To reproduce neutral pH conditions the side chains of aspartates and glutamates were negatively charged, those of lysines and arginines were positively charged, and histidines were considered neutral. The protein was immersed in an orthorhombic box of preequilibrated water molecules. The size of the box was chosen to have a minimal distance of 13 Å between the boundary and any atom of the protein. Solvent molecules within 2.4 Å of any heavy atom of the protein were removed except for six water molecules present in the crystal structure. The simulation system contained 8 sodium and 9 (10 for the DAP) chloride ion to compensate for the total charge of FKBP which is +1 electron units. The MD simulations were carried out with NAMD [31] using the CHARMM22 force field [32] and the TIP3P model of water [33]. The parameters of the six ligands were determined according to the general CHARMM force field [34]. Periodic boundary conditions were applied and electrostatic interactions were evaluated using the particle-mesh Ewald summation method [35]. The van der Waals interactions were truncated at a cutoff of 12 Åand a switch function was applied starting at 10 Å. The MD simulations were performed at constant temperature (310 K or 350 K) using the Langevin thermostat and constant pressure (1 atm) [36] with a time step of 2 fs. The SHAKE algorithm was used to fix the covalent bonds involving hydrogen atoms.

For each ligand and temperature value, 50 independent MD runs were carried out with different initial velocities. The runs were stopped after 20 ns or before if the intermolecular distance exceeded 30 Å. The Cartesian coordinates were saved every 4 ps along the trajectories. Thus, the number of snapshots used for analysis is different for different ligands, and ranges from 109569 for DMSO to 169511 for DSS.

### Analysis of MD simulations and clustering procedure

The analysis of the MD trajectories was carried out with CHARMM [37] and the MD-analysis tool WORDOM [38]. The leader algorithm as implemented in the latter program was employed for clustering according to the distance root mean square between two MD snapshots a and b, DRMS 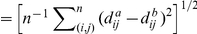, which was calculated using the intermolecular distances 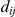 between pairs of non-hydrogen atoms in the ligand and eight residues in the FKBP active site (Tyr26, Asp37, Phe46, Val55, Ile56, Trp59, Tyr82, and Phe99). A DRMS threshold of 1 Å was used for clustering by the leader algorithm. The complex network analysis (see below) and cut-based FEP (see Fig. S22 in Text S1) are robust with respect to the choice of the DRMS threshold in the range 0.8 to 1.0 Å. The DRMS calculation does not require structural overlap. In other words, rigid-body fitting is not necessary, which is an advantage with respect to the root mean square deviation.

### Construction of the unbinding network of BUT

The clustering of about 150000 MD snapshots of BUT (35 runs of 10 ns, and 15 runs of 15–20 ns) yielded 18021 clusters with two or more snapshots and 11425 one-snapshot clusters. The 29446 clusters are the nodes of the network and the transitions between them are edges. Note that the terms node and cluster are used as synonyms in this work. Totally there were 73473 edges within nodes and 74801 edges between different nodes. The networks were plotted using a spring-embedder algorithm [39] as implemented in the program igraph (cneurocvs.rmki.kfki.hu). The overall features of the network are robust with respect to the choice of the thresholds on link and node size. Moreover, it is important to note that the clustering was not used for the analysis of unbinding kinetics but only for plotting the network and the cut-based FEP. The unbinding times were extracted directly from the MD trajectories without using the clustering.

### Cut-based FEP

**Figure 1:**
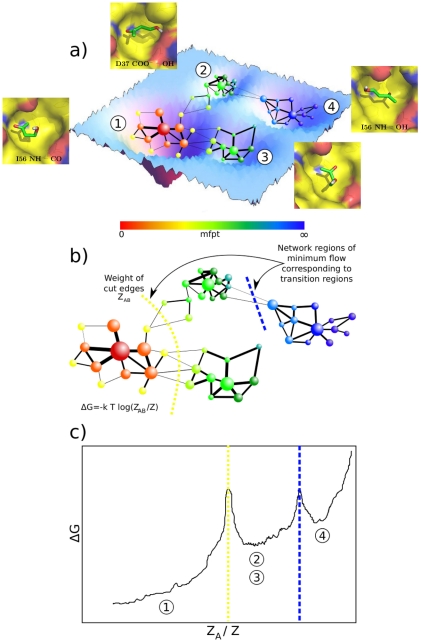
Illustration of the cut-based FEP [**13**]. (a) The high-dimensional free-energy surface is coarse-grained into nodes of the network. Two nodes are linked if the system proceeds from one to the other along the considered timeseries. The mean first passage time (mfpt) is calculated for each node analytically (see text). (b) For each value of mfpt the set A of all nodes with a lower mfpt value is defined. The free-energy 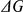 of the barrier between the two states formed by the nodes in A and the remainder of the network B can be calculated by the number of transitions 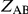 between nodes of either set [13]. (c) The cut-based FEP is a projection of the free-energy surface onto the relative partition function 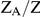, which includes all pathways to the reference node. For each value of mfpt, the point 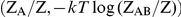 is added to the FEP. The cut-based FEP projects the free-energy surface faithfully for all nodes to the left of the first barrier (basin 1). After the first barrier, two or more basins overlap (e.g., basins 2 and 3) if they have the same kinetic distance from the reference node.

Projected free-energy surfaces are most useful if they preserve the barriers and minima in the order that they are met during the sequence of events. Krivov and Karplus have exploited an analogy between the kinetics of a complex process and equilibrium flow through a network to develop the cut-based FEP, a projection of the free energy surface that preserves the barriers [13] and can be used for extracting folding pathways and mechanisms from MD simulations [21]. The input for the cut-based FEP calculation is the transition network, which is derived by clustering, e.g., as described above. For each node 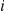 in the transition network, the partition function is 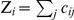, i.e., the number of times the node 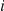 is visited, where 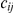 is the number of direct transitions from node 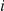 to node 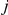 observed along the time series. The transition probabilities can then be calculated as 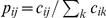. If the nodes of the transition network are partitioned into two groups A and B, where group A contains the reference node, then 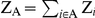 (the number of times a node in 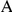 is visited), 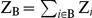, and 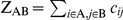 (the number of transitions between nodes in 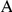 and nodes in 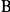). The free energy of the barrier between the two groups is 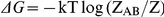, where 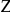 is the partition function of the full transition network (Fig. 1). The progress coordinate then is the normalized partition function 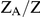 of the reactant region containing the reference node, but other progress coordinates can be used, because the cut-based FEP is invariant with respect to arbitrary continuously invertible transformations of the reaction coordinate [40].

In practice, the procedure to calculate the cut-based FEP consists of three steps (Fig. 1): (1) The mean first passage time (mfpt) of node 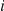 to the reference-node is the solution of the system of equations mfpt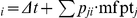 with initial boundary condition mfpt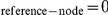 [41]. The timestep 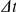 corresponds to the saving frequency of 4 ps; i.e., the mfpt of a node is defined as one timestep plus the weighted average of the mfpt values of its adjacent nodes. (2) Nodes are sorted according to increasing values of mfpt (or decreasing values of the probability of binding); for each value of the progress variable the relative partition function 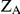 and the cut 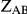 are calculated. (3) The individual points on the profile are evaluated as (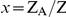, y = 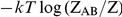). The cut-based FEP method has been applied to characterize the free energy surface and folding pathways of the 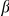-hairpin of protein G [13], a three-stranded antiparallel 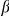-sheet peptide [21], [22], and a cross-linked 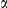-helical peptide [25]. Recently, the cut-based FEP analysis of a simplified model of an amphipathic aggregation-prone peptide has provided strong evidence that amyloid fibril formation is under kinetic control [42].

Detailed balance was imposed to the network, i.e., the number of transitions from node 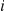 to node 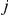 (and vice versa) was set equal to the arithmetic mean of the transitions from 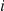 to 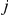 and from 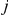 to 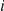. Such symmetrization of the transition network improves the statistics and introduces a negligible error in the bound state since the trajectories are much longer than the slowest relaxation time within the bound state.

Moreover, for each fragment several rebinding events were observed along the MD runs, so that the sampling of the dissociation barrier is at local equilibrium. The mfpt and the cut-based FEPs were calculated by the program WORDOM [38] using, as mentioned above, a time interval of 4 ps. The cut-based FEPs were also evaluated using the same DRMS clustering but taking into account MD snapshots saved with a time interval of 8 ps (see Fig. S23 in Text S1) to check that the clustering procedure preserves the diffusive behavior of the dynamics [40]. This test is a necessary (though not sufficient) condition for the appropriateness of the clustering because the dynamics of spontaneous ligand unbinding is expected to be in the diffusive regime.

### Probability of unbinding and transition state identification

The probability of unbinding can be evaluated for each MD snapshot very efficiently by considering that all snapshots in a node have the same probability of unbinding as described originally for the probability of folding [43]. The basic assumption is that conformations that are structurally similar have the same kinetic behavior, hence they have similar unbinding probability [22], [43]. The MD trajectory following a given snapshot is analyzed to check if the unbinding condition is satisfied within a commitment time that has to be chosen much shorter than the unbinding time. An unbinding event is defined by a separation between the centers of mass of the FKBP active site and the ligand larger than 15 Å. For each node, the unbinding probability is the ratio between its members that unbind and the total number of snapshots in the node. The node with unbinding probability between 0.45 and 0.55 are defined as the transition state ensemble (TSE). Among these, only those with at least 20 MD snapshots were taken into account.

## Results

Starting from the bound conformation with the ligand in the active site of FKBP [30], 50 independent MD runs at 310 K, as well as 50 runs at 350 K presented mainly in the SI, were carried out for each of the six ligands of FKBP (Table 1). Each run has a length between 10 and 20 ns (as the simulations were not elongated when the intermolecular distance exceeded 30 Å), and the cumulative simulation time for the six ligands and two temperature values is about 10 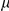
s. The FKBP structure was remarkably stable in all MD runs: the C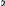 root mean square deviation from the X-ray structure is 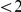 Å for 95% of the snapshots at 310 K and for 79% of the snapshots at 350 K. Moreover, only 0.1% and 1% of the snapshots at 310 K and 350 K, respectively, have a C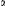 root mean square deviation larger than 3 Å (and smaller than 4 Å). Most of the analysis focusses on BUT while the networks and kinetic analysis of the other five ligands are presented in the SI.

**Table 1:** The six ligands of FKBP sorted according to binding affinity.

Compound	Unbinding^a^ events	Rebinding^b^ events	Binding affinity (LIE model)^c^			Unbinding^d^	Experimental^e^ value of K 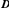 (mM)
			vdWaals	electr.	Total	time	
				(kcal/mol)		(ns)	
DMSO	49	5	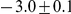	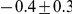	-3.4	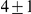	20.0
PENT	34	10	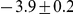	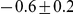	-4.5	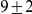	2.0
BUT	40	8	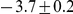	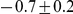	-4.4	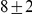	0.5
DAP	45	12	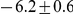	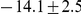	-20.3	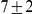	0.5
DSS	29	9	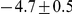	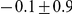	-4.8	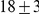	0.25
THI	32	10	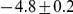	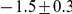	-6.3	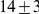	0.2

The six ligands are: BUT, 4-hydroxy-2-butanone; DMSO, dimethylsulfoxide; DAP, 5-diethylamino-2-pentanone; DSS, methyl sulphinyl-methyl sulphoxide; PENT, 5-hydroxy-2-pentanone; THI, tetrahydrothiophene 1-oxide.

a An unbinding event is defined as a separation of the ligand center of mass from the center of the FKBP binding site larger than 15 Å.

b A rebinding event is defined as an unbinding event followed by a separation of the ligand/FKBP binding site smaller than 10 Å.

c The binding affinity in the LIE model is approximated as the difference of the interaction energy of the ligand with two different surroundings, the protein and solvent in the bound state and only the solvent in the unbound state [50]. The LIE binding energy is calculated by averaging separately over all bound or unbound conformations using a cutoff of the intermolecular distance of 15 Å to discriminate between bound and unbound. The electrostatic energy term is multiplied by 0.5 to be consistent with the hydration energy of a single ion, which is equal to half the corresponding ion-water interaction energy [54]. The van der Waals energy term is multiplied by an empirical parameter 0.56 which is derived from linear fitting using only the five neutral compounds. Each of the total sampling is divided into five blocks and the block averaging errors for both energy terms are given in the table.

d The unbinding time 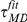 is calculated by a single exponential fit of the cumulative distribution of the unbinding events detected along the MD trajectories (see Fig. 5). The unbinding time and error for each ligand are evaluated by single-exponential fitting using 25 randomly selected MD runs out of 50, and calculating the average error for the remaining 25 MD runs not used for the fitting, i.e., the difference between the value predicted by the fitting curve and the unbinding time measured along the MD trajectory. This procedure is repeated 100 times for each ligand, and average values of unbinding time and cross-validated error are reported in the table.

e Measured by a fluorescence assay [30].

### MD simulations of spontaneous unbinding

**Figure 2:**
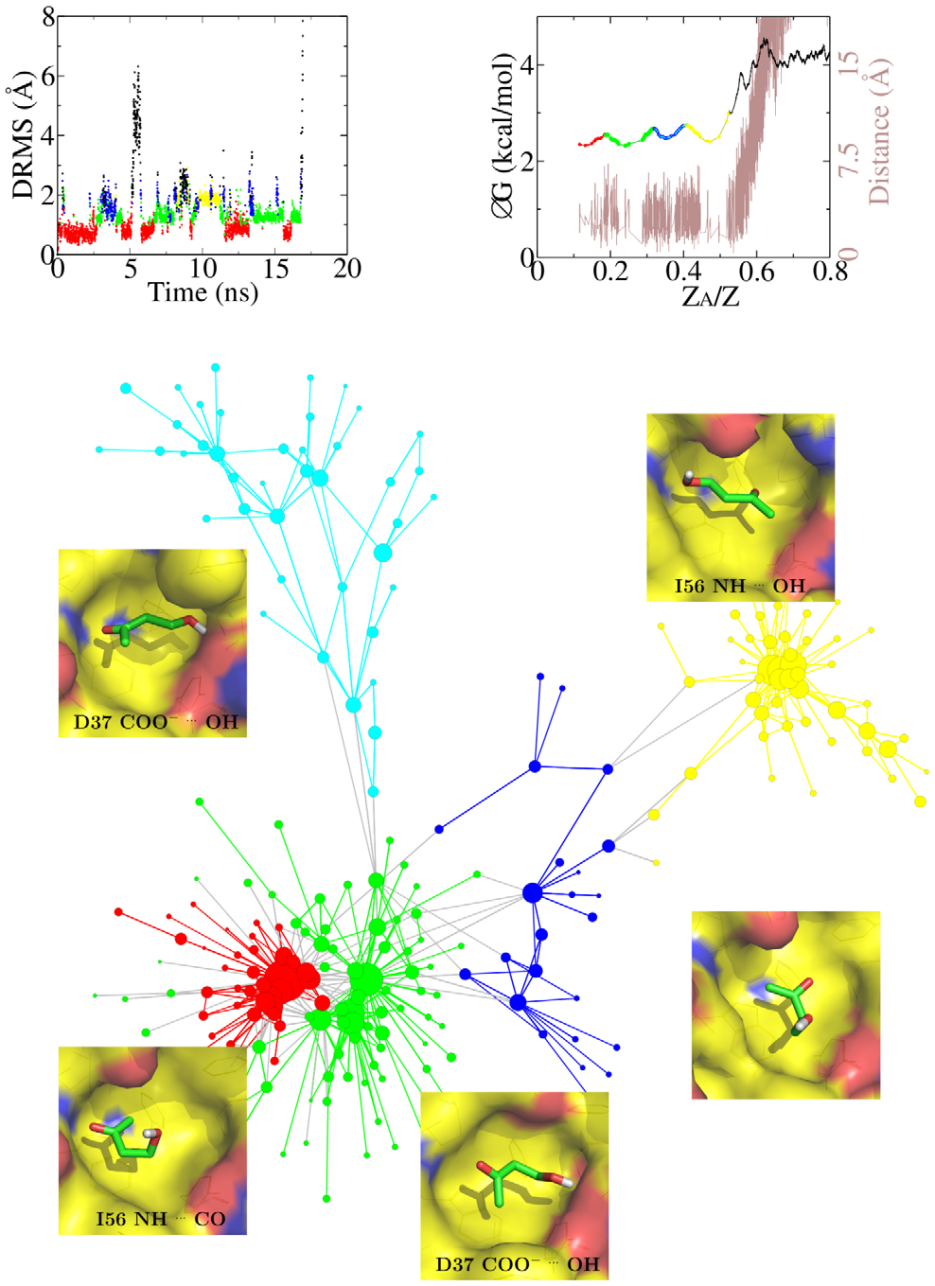
Multiple binding modes of BUT. The binding modes of BUT in the active site of FKBP, i.e., the subbasins within the bound state, were determined by the cut-based FEP approach [13] and are shown by different colors. (Top,left) Time series of DRMS from the X-ray structure of the BUT/FKBP complex [30] for one of the 50 MD runs at 310 K. The majority of MD snapshots in the most populated subbasin (red) have a DRMS smaller than 1.0 Å. The interconversions between subbasins are evident. The time series of other 20 MD runs are shown in Fig. S1 in Text S1. (Top,right) Cut-based FEP at 310 K and distance between centers of mass of BUT and FKBP active site with y-axis on the left and right, respectively. The most populated node is employed as reference, and the relative partition function Z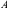/Z is used as reaction coordinate as it takes into account all routes from the reference state [13]. The cyan and blue nodes overlap in the third subbasin from the left because they have the same kinetic distance from the reference node. (Bottom) Network representation [11] of the bound state of BUT. Nodes and links are the conformations (i.e., clusters obtained by DRMS clustering) and direct transitions (i.e., within 4 ps), respectively, sampled in the 50 MD runs at 310 K. The size of each node is proportional to the natural logarithm of its statistical weight, and only nodes connected by at least one link of weight 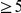 are shown to avoid overcrowding. Links connecting pairs of nodes in the same subbasin have the same color of the subbasin, otherwise they are gray. In the insets close to each basin, the FKBP surface is colored according to atom type with carbon atoms surface in yellow while BUT is shown by sticks with carbon atoms in green.

In the majority of the runs the ligand separates completely from the surface of FKBP (Fig. 2,top, see also Figs. S1 and S2 in Text S1). The ligand with the lowest affinity, DMSO, shows the highest number of unbinding events (49 in the 50 MD runs), while the two ligands with highest affinity, THI and DSS the smallest number (32 and 29, respectively, Table 1). The number of rebinding events ranges from 5 for DMSO to 12 for DAP (Table 1 and see Fig. S2 in Text S1). Since there are many more unbinding events than rebinding events the analysis focusses on unbinding kinetics and the relative probabilities of the binding modes.

The dissociation rates, extracted for each ligand by fitting the cumulative distribution of the unbinding events observed in the 50 MD runs (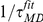, see subsection Multiple unbinding pathways and single-exponential kinetics of unbinding), show a Pearson correlation coefficient of −0.84 with the equilibrium dissociation constants measured by a fluorescence assay [30] (see Fig. S3 in Text S1). Since the dissociation constant is the ratio between the off-rate and the on-rate the correlation indicates that the on-rate might be similar for the six ligands considered in this study.

The residence time of a ligand on a protein surface or cavity can be measured by NMR spectroscopy or surface plasmon resonance. Experimentally, the residence time varies from picoseconds for very small ligands, e.g., water and urea [44], [45], [46], [47], to milliseconds and seconds for potent binders, like high affinity inhibitors and antibodies [48], [49]. The six small ligands of FKBP considered in the present study have intermediate size and affinity so that their unbinding times in the nanosecond time scale are consistent with the residence times measured experimentally for smaller and larger molecules.

### Energy contributions to binding affinity

It is not possible to calculate the free energy of binding directly from the populations of bound and unbound as the MD runs where stopped upon ligand dissociation so that the relative populations are not correct. Therefore, the linear interaction energy (LIE) model [50] is used to approximate the binding energy as 
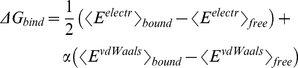
(1)


where 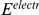 and 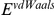 are the electrostatic and van der Waals interaction energies between the ligand and its surroundings, respectively. The 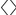 denotes an ensemble average sampled over a MD [51] or Monte Carlo [52] trajectory. Here, each of the two non-bonding terms is averaged independently over the trajectory segments during which the ligand is bound (ligand/protein plus ligand/water interactions) and the segments when the ligand is fully dissociated (ligand/water interactions). The coefficient 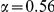 is determined empirically [51] by linear fitting using the five neutral compounds. The multiplicative factor 1/2 for the electrostatic term has a physical justification which can be explained by the fact that the electrostatic contribution to the hydration energy of a single ion is equal to half the corresponding ion-water interaction energy [53], [54]. One advantage of the LIE model is that the two non-bonding energy terms can be analyzed individually. For the five neutral ligands the values of the binding affinity (in the LIE approximation) span a relatively small range, from 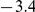 kcal/mol for DMSO to 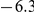 kcal/mol for THI, and the van der Waals term has a more favorable contribution than the electrostatic term (Table 1). In contrast, the LIE binding affinity of DAP is much more favorable (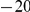 kcal/mol) and is dominated by the electrostatic energy because of the salt bridge between the Asp37 side chain and the tertiary amino group of DAP which is positively charged. Therefore, the binding affinity in the LIE model is not a good approximation of the free energy of binding particularly for charged compounds for which polarization effects [55] are neglected in force fields with fixed partial charges. In addition, the electrostatic desolvation penalty depends strongly on the water model used in the simulations, which has a much stronger influence on charged species than neutral.

### Multiple binding modes

Analysis of the MD trajectories reveals that multiple binding modes in the active site of FKBP are sampled for all six ligands (Fig. 2 and see Figs. S4–S15 in Text S1). Interestingly, the electron density maps indicate that PENT and DAP are present in the soaked FKBP crystals, but the quality of the maps was poor so that the crystallographers stated that “it is likely that these rather flexible ligands bind in a number of different conformations” [30]. Other computational and experimental studies have also reported and analyzed multiple binding modes [56], [57], [58].

**Figure 3:**
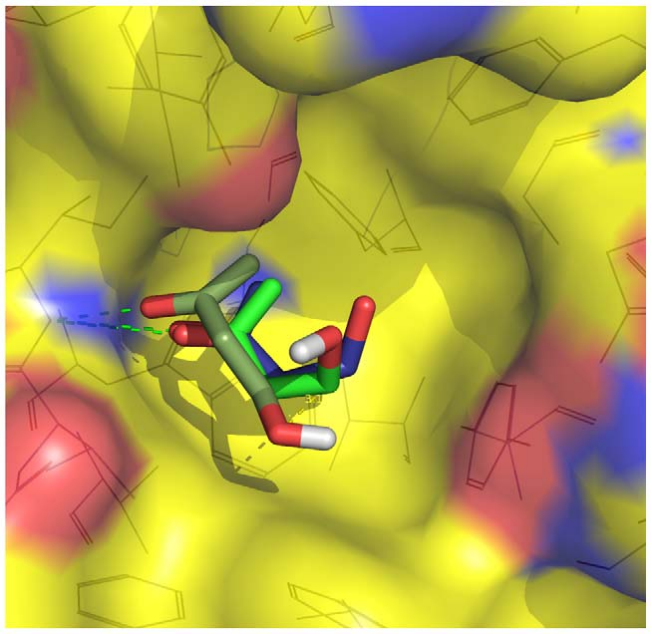
The binding mode observed most frequently in the MD simulations corresponds to the one in the X-ray structure. Two binding poses of BUT from the red subbasin (carbon atoms in green) are shown together with the pose of BUT in the crystal structure (carbon atoms in blue) upon optimal overlap of the C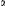 atoms of FKBP. The surface of FKBP is colored according to atom type with carbon, oxygen, and nitrogen atoms in yellow, red, and blue, respectively. The hydrogen bond between the NH of Ile56 and the carbonyl oxygen of BUT is shown by green dashed lines.

It is useful to focus on BUT because it is one of the three ligands (the other two are DMSO and DSS) for which the X-ray structure in the complex with FKBP has been solved [30]. The ligand BUT has two hydrogen bond acceptors and one donor, the carbonyl and hydroxyl groups, separated by two methylene groups. It either accepts a hydrogen bond from the amide nitrogen of Ile56 or donates a hydrogen bond to the side chain of Asp37 as the distance between the two polar groups of BUT is not long enough to allow for the simultaneous formation of both intermolecular hydrogen bonds. The network analysis [11] and FEP [13] consistently reveal multiple subbasins in the bound state of BUT (Fig. 2) as well as for the other ligands (See Figs. S4 and S5 in Text S1). The red and green subbasins make up about 50% of the number of snapshots of the bound conformation of BUT, and the binding mode of BUT with its carbonyl group acting as acceptor for the NH of Ile56 (red subbasin) is identical to the one in the X-ray conformation (Fig. 3). There is also an end-to-end flipped orientation of BUT in which its hydroxyl group (instead of the carbonyl) accepts from the NH of Ile56. This pose makes up the subbasin of yellow nodes, which include about 25% of total bound conformations. The energy barriers between poses in different subbasins are small, which allows fast interconversions as observed in the time series of DRMS deviation from the X-ray structure (Fig. 2). There are more jumps between green and red subbasins than between green/red and yellow as the latter transitions require an end-to-end flip of BUT.

### Multiple unbinding pathways and single-exponential kinetics of unbinding

**Figure 4:**
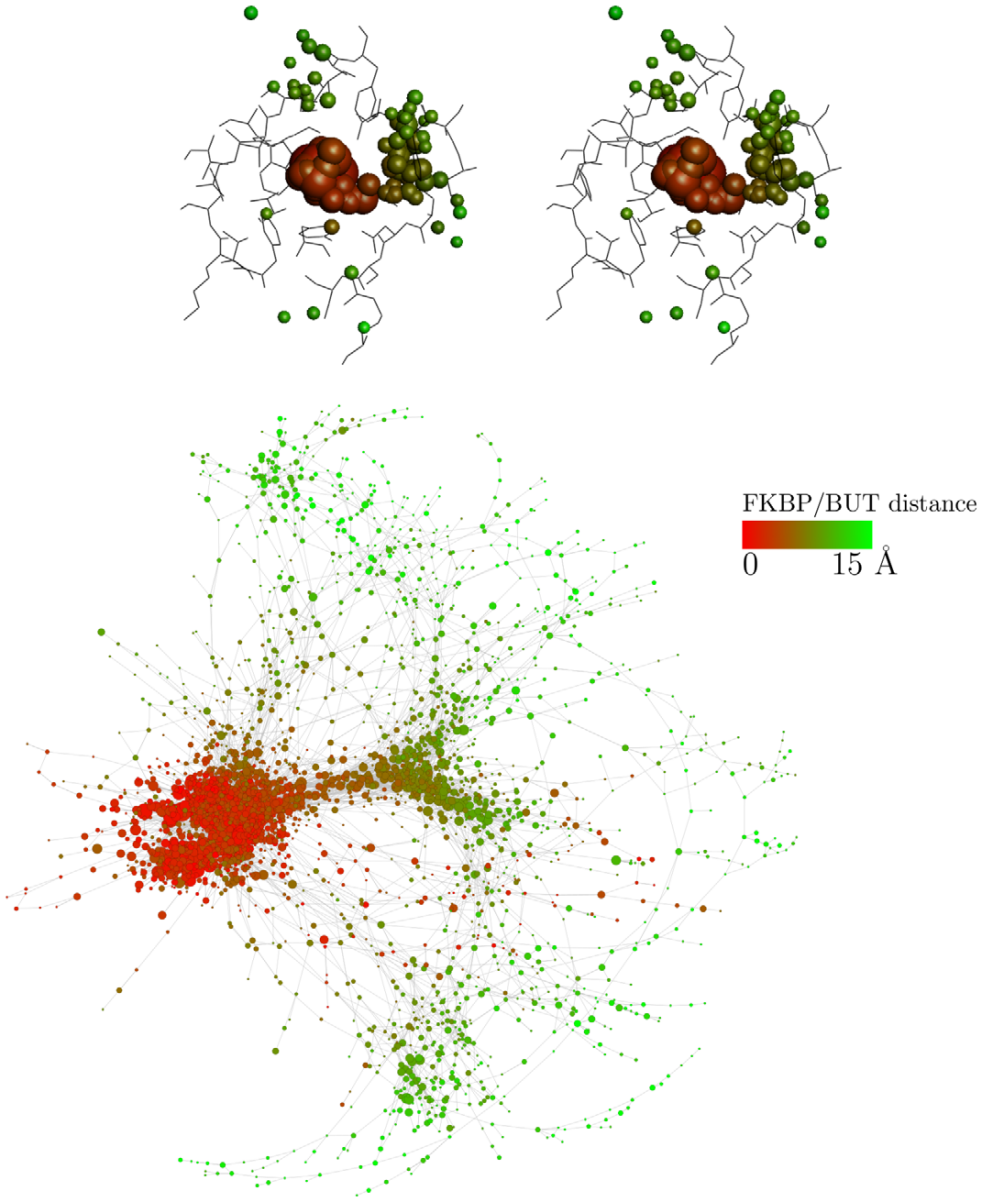
Multiple unbinding pathways. The red/green coloring illustrates the distance between centers of mass of BUT and FKBP active site. To illustrate the unbinding pathways, all frames of the 50 MD runs are first overlapped in space [66] using the coordinates of the C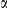 atoms of FKBP. The different positions and orientations of BUT are then clustered according to DRMS with a threshold of 1 Å. (Top) Stereoview of the most populated clusters. The radius of the spheres is proportional to the natural logarithm of the corresponding cluster population. (Bottom) Ligand unbinding network colored according to the distance between BUT and FKBP. Nodes and links are the clustered conformations and direct transitions, respectively [11]. The size of each node is proportional to the natural logarithm of its statistical weight. Only the 4184 nodes with distance between the centers of mass of the ligand and FKBP active site smaller than 15 Å were taken into account; of these, only the 2918 nodes with at least two MD snapshots are shown to avoid overcrowding. Nodes of the bound state, i.e., those in Fig. 2, bottom, are all included in the dense region of red nodes on the left.

**Figure 5:**
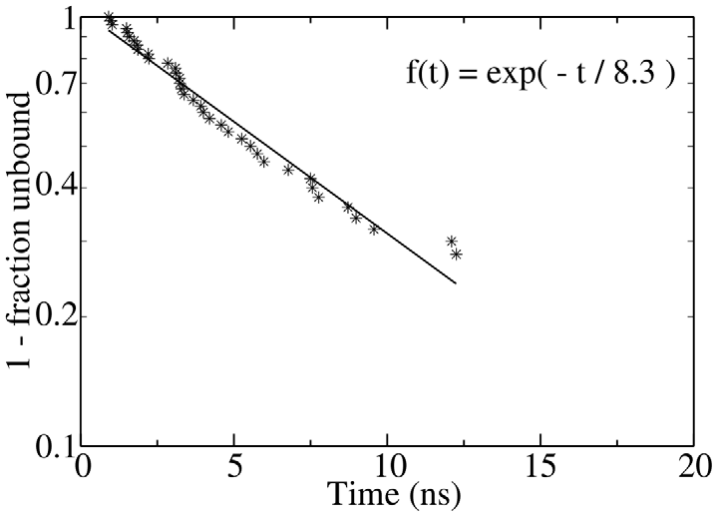
Single-exponential kinetics of unbinding. The plot shows the cumulative distribution 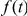 of the unbinding times; 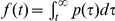, where 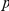 is the probability distribution of the unbinding time. An unbinding event is defined by a separation between the centers of mass of the FKBP active site and the ligand larger than 15 Å. The stars represent the 40 unbinding events observed in the 50 MD runs of BUT. The single-exponential fit (solid line) yields 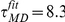 ns.

The time series of DRMS show that in most trajectories of BUT there are several interconversions between different binding modes, which take place before the event of total dissociation (Fig. 2). In addition, the network analysis illustrates that there are different unbinding pathways without a single predominant route (Fig. 4). The unbinding pathways are spread over a large section of the active site and/or its rim (see also subsection Unbinding transition state and Hammond effect). Despite the multiple pathways of unbinding, the cumulative distribution of the unbinding time shows single-exponential behavior (Fig. 5). Given that equilibration within the bound state is much faster than unbinding (the time series in Fig. 2 top, left shows that multiple interconversions between bound state subbasins take place before unbinding), the single-exponential kinetics suggests that different pathways of dissociation have similar barrier height. Note that the multiple interconversions within the bound state, multiple pathways of dissociation, and single-exponential time dependence of the unbinding kinetics are observed for all six ligands (see Figs. S1, S16–S19 in Text S1).

The observation that the unbinding barrier is much higher than the barriers between subbasins suggests that, at least for small and low-affinity ligands, the starting pose does not influence the unbinding simulation results. To provide additional evidence to this observation, 10 conformations in the bound state of DMSO were randomly chosen from the 50 MD simulations, and 10 runs at 310 K with different initial velocities were started for each of them. In another test, 50 runs with different initial velocities were started for each of five randomly oriented poses of DMSO in the active site of FKBP. The 250 simulations of the second test were carried out at 350 K to speed up the sampling. The unbinding times (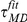 values) derived from simulations using different starting conformations of DMSO are very similar among each other (see Figs. S20 and S21 in Text S1).

The unbinding network and cut-based FEP at 350 K are qualitatively similar to those extracted from simulations at 310 K and reveal multiple binding modes. The main difference is that the dissociation kinetics are faster as the unbinding barriers are lower at 350 K than 310 K (See Fig. S3 in Text S1), which is consistent with the mainly enthalpic nature of the dissociation barrier.

### Unbinding transition state and Hammond effect

**Figure 6:**
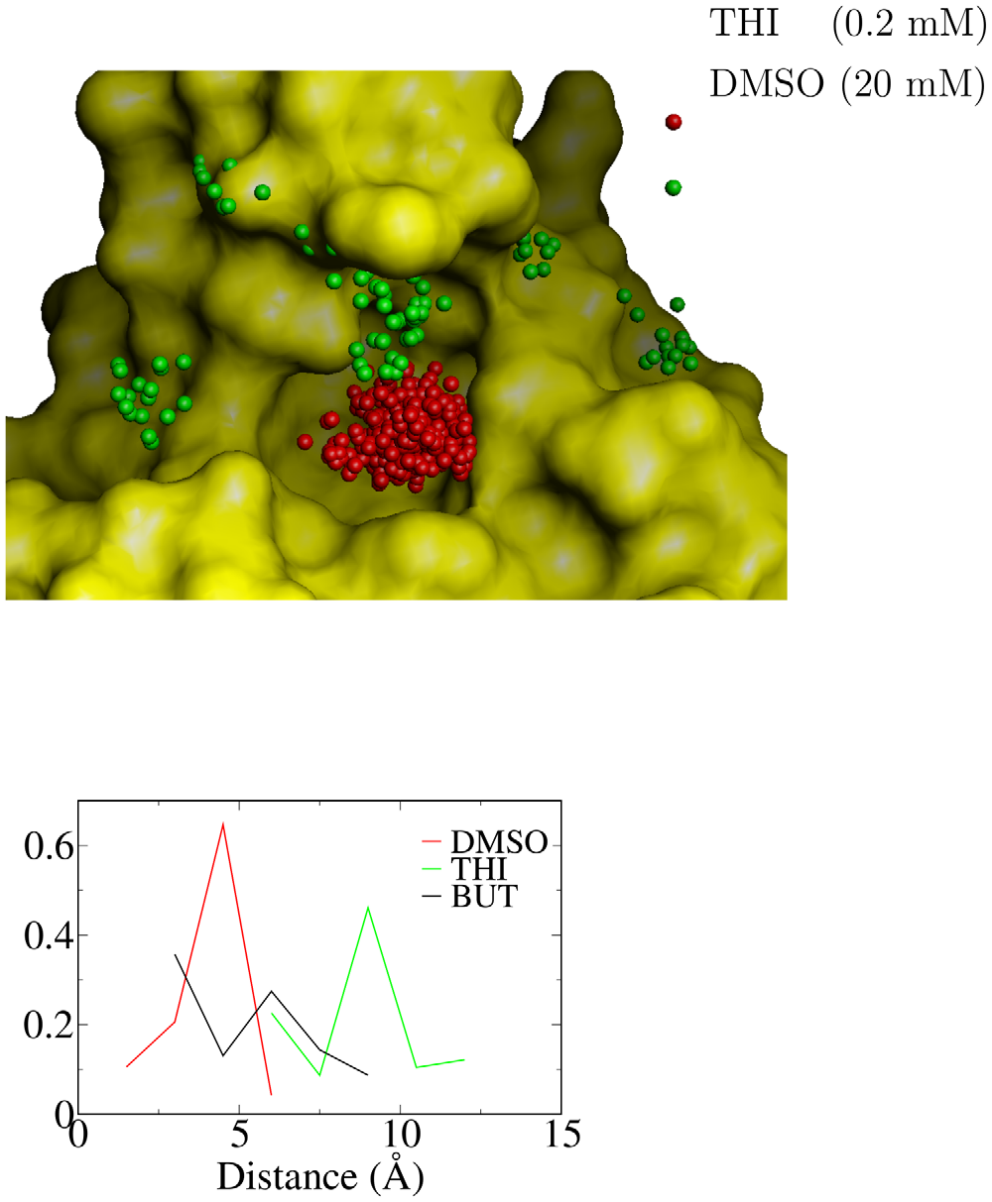
Unbinding TSE and Hammond behavior. The structures belonging to the TSE were identified along the MD trajectories by a procedure based on the probability to unbind within a commitment time [22], [43]. A commitment time of 0.8 ns was used for all ligands, and individual conformations were assigned to the TSE if their unbinding probability was in the 0.45 to 0.55 range. (Top) The surface of FKBP is shown in gold while the positions of the centers of mass of the ligands at the TSE are shown by spheres. (Bottom) Distribution of distance between centers of mass of ligand and FKBP active site at the TSE. Note that the Hammond behavior, i.e., the shift of the TSE along the unbinding reaction coordinate, is robust with respect to the choice of the commitment time (Table S1 in Text S1).

The probability to unbind can be defined analogously to the probability of folding [59], [60]. For each ligand, the TSE is determined along the 50 MD trajectories by a procedure based on the probability to unbind within a certain commitment time [22], [43]. Values of 0.45 to 0.55 for the probability to unbind and commitment time of 0.8 ns are used, and the robustness of the TSE on these choices is documented in Table S1 in Text S1. The unbinding TSE consists of a broad variety of positions and orientations of the ligand in the FKBP active site and/or at its rim (Fig. 6,top). The heterogeneity of the TSE, and in particular the broad distribution of TSE structures over the whole surface of the active site, is consistent with the multiple unbinding pathways detected by the network analysis.

For ligands with different values of the dissociation rate (and affinity) it is interesting to compare the position of the TSE along the reaction coordinate of unbinding. The distance between the centers of mass of ligand and FKBP active site can be used for this analysis as it is an intuitive geometric coordinate and a good predictor of the mfpt to the most populated node (Pearson correlation coefficient higher than 0.90 up to distances of 30 Å). Despite the relatively small difference in affinity for FKBP of only a factor of about 100, the TSE of DMSO is shifted with respect to the one of THI along the center of mass distance towards the state that is destabilized, i.e., the bound state (Fig. 6). The TSE conformations of THI is located mainly at the rim of the active site which might be due in part to its additional van der Waals interactions with FKBP as THI has two more carbon atoms than DMSO. An intermediate shift is observed for BUT (Fig. 6,bottom) and the other four ligands (Table S1 in Text S1) which is consistent with their values of the dissociation constant being between those of THI and DMSO. Note that the shift is not due to the different sizes and number of atoms of the ligands because there is no correlation between TSE shift and size (Table S1 in Text S1). The TSE shift is a manifestation of the Hammond effect, which was described 55 years ago for chemical reactions: As the substrate (here the ligand-bound state) becomes more unstable, the transition state approaches it in structure [61]. A shift of the protein folding TSE in the direction of the destabilized state has been observed previously upon single-point mutations in small, single-domain proteins [62]. On the other hand, Hammond behavior has not been reported for ligand (un)binding.

## Discussion

Five main results emerge from the network and cut-based FEP analyses of the MD simulations of unbinding of six small ligands from the active site of FKBP. First, fully atomistic simulations of spontaneous ligand unbinding from the active site of an enzyme are computationally feasible. The MD trajectories can be used to characterize the free energy surface of the bound state and the unbinding kinetics. Second, both the network analysis and cut-based FEP method reveal that each ligand has multiple poses (characterized by distinct intermolecular hydrogen bonds) in the bound state. Moreover, unbinding proceeds through multiple pathways. A similar free energy landscape with multiple pathways was previously observed in equilibrium simulations of the reversible folding of structured peptides [21], [23] and small proteins [27], [63], [64]. Third, the kinetics of small ligand dissociation from FKBP are simple and their time dependence can be fitted by a single-exponential function despite the presence of multiple binding modes and multiple exit pathways. The rate-limiting step of unbinding is characterized by a free energy barrier that is much higher than the barriers between subbasins (i.e., binding modes) in the bound state. Fourth, the unbinding TSE consists of a broad variety of ligand poses which lead to multiple dissociation pathways. Finally, a comparative analysis of the TSE of the six ligands shows that the smaller the stability of the bound state the closer are the TSE poses to the bound structure which is a new example of Hammond behavior, i.e., shift of the TSE towards the destabilized state.

It is likely that some of the conclusions of this work are valid also for drug-like compounds, which are larger (20 to 50 non-hydrogen atoms) and more potent (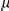
M to nM affinity) than the six ligands investigated here. In particular, multiple (un)binding pathways are likely to exist also for high-affinity ligands, even if they usually have a single binding mode. Using network analysis and the cut-based FEP method it might become possible in the future to investigate ligands of nM affinity, which will require about one to two orders of magnitude longer simulations. This estimation is based on the aforementioned linear fitting of natural logarithm of unbinding times of the six ligands of FKBP to their experimentally measured binding energy values (See Fig. S3 in Text S1), which yields an extrapolated unbinding time of about 200 ns for a 200 nM ligand. In this context, it is important to note that small fragments used in the early phase of drug discovery bind usually in the mM to 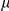
M range. Another interesting application could be the analysis of the free energy landscape of binding of small molecules with very similar chemical structure but significantly different binding affinity, e.g., a series of protein kinase inhibitors that differ by only one to two heavy atoms and whose affinity ranges from micromolar to single-digit nanomolar [65].

## Supporting Information

Text S1
**This file contains the supporting figures and table for this article.**

**Figure S1:** Time series of DRMS from the X-ray structure for 20 of the 50 runs of BUT at 310 K. The y axis is DRMS in Å and x axis is time in ns. **Figure S2:** Time series of distance between centers of mass of BUT and FKBP active site in 20 of the 50 runs at 310 K. The y axis is distance in Å and x axis is time in ns. The green or red line indicates distance at 15 or 10 Å. **Figure S3:** Scatter plot of experimental binding energies versus natural logarithm of the unbinding times extracted from MD at 310 and 350 K. The Pearson correlation coefficient is −0.84 and −0.83 for 310 and 350 K MD runs, respectively. The unbinding time and error for each ligand are evaluated by single-exponential fitting of the cumulative distribution function of unbinding times using 25 randomly selected MD runs out of 50, and calculating the average error for the remaining 25 MD runs not used for the fitting, i.e., the difference between the value predicted by the fitting curve and the unbinding time measured along the MD trajectory. This procedure is repeated 100 times for each ligand, and average values of unbinding time and cross-validated error are shown. **Figure S4:** Cut-based FEPs of six ligands at 310 K (black). The distance between centers of mass of ligand and FKBP active site (green) and the mean first passage time (red) are also shown with y-axis on the right. **Figure S5:** Network representation of the bound states of the six ligands at 310 K. The largest 25 nodes are marked with numbers and their representatives are shown in Fig. S6–S11 in Text S1. **Figure S6:** Representative poses of the largest 25 nodes of DMSO. **Figure S7:** Representative poses of the largest 25 nodes of PENT. **Figure S8:** Representative poses of the largest 25 nodes of BUT. **Figure S9:** Representative poses of the largest 25 nodes of DAP. **Figure S10:** Representative poses of the largest 25 nodes of DSS. **Figure S11:** Representative poses of the largest 25 nodes of THI. **Figure S12:** Cut-based FEPs plotted using as reference node the most populated node of individual subbasins. These cut-based FEPs were used to determine the subbasins of the bound state. The cut-based FEP on the top left corresponds to the one in Figure 2 of the main text. **Figure S13:** Simplified network of subbasins in the bound state of BUT. The nodes are the subbasins identified with the procedure shown in Fig. S12 in Text S1 except for the black node which represents the unbound state. The thickness of the links is proportional to the number of the transitions observed in the 50 MD runs at 310 K. **Figure S14:** Network representation of the bound states of the six ligands at 350 K. Only nodes connected by links of weight 5 or more are shown to avoid overcrowding. **Figure S15:** Cut-based FEPs of six ligands at 350 K. **Figure S16:** Single-exponential kinetics of unbinding for 6 ligands at 310 K. The plots show the cumulative distribution f(t) of the unbinding times observed in the 50 MD runs. Note that the unbinding times obtained by fitting are slightly different from those in Table 1 of the main text because a cross-validation procedure was used in the latter. **Figure S17:** Single-exponential kinetics of unbinding for 6 ligands at 350 K. The plots show the cumulative distribution f(t) of the unbinding times for 6 ligands at 350 K. The unbinding times range from 1.6 to 5.6 ns, which is shorter than the corresponding values at 310 K. **Figure S18:** Network representations of the bound state for DMSO (top left), PENT (top right), BUT (middle left), DAP (middle right), DSS (bottom left), and THI (bottom right). Nodes are colored from red to green according to the distance of the centers of mass of ligand and FKBP. **Figure S19:** Stereoview of the most populated clusters for 6 ligands - DMSO, PENT, BUT, DAP, DSS and THI (top to bottom). Nodes are colored from red to green according to the distance of the centers of mass of ligand and FKBP. **Figure S20:** Test at 310 K with DMSO. Ten bound state conformations were randomly chosen from previous MD simulations and ten runs of 10 ns each with different initial velocities were started for each of them. Single-exponential kinetics of unbinding is observed and the unbinding time derived from the plot is 4.2 ns which is similar to the value derived from the 50 runs started from the X-ray structure of the complex. **Figure S21:** Test at 350 K with DMSO. Fifty 5-ns runs with different velocities were started for each of five randomly oriented poses of DMSO in the active site of FKBP. Single-exponential kinetics of unbinding is observed and the unbinding times derived from the plots range from 1.3 to 1.9 ns, which is consistent with the value derived from the 50 runs started from the X-ray structure of the complex (top, left). **Figure S22:** The cFEPs for DMSO (left) and PENT (right) were obtained using DRMS clustering cutoffs of 0.8 Å, 0.9 Å, 1.0 Å, and 1.5 Å from top to bottom. **Figure S23:** Diffusivity test for the clustering of DMSO and THI. The profiles with saving frequency at 4 and 8 ps are similar upon a vertical shift of ln(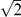), which is consistent with the diffusive regime. **Table S1:** Robustness of TSE definition and Hammond behavior. Each column lists the average distances between the centers of mass of the ligand and FKBP active site for the conformations at the TSE. The numbers of TSE nodes and snapshots are shown in parentheses. Only TSE nodes with weight larger than 5 were used for this analysis as nodes with very low weight increase the noise.
**Note:** A movie of the MD simulation of spontaneous unbinding of BUT from FKBP can be found at http://www.biochem-caflisch.unizh.ch/movie/7/.(PDF)
