# Supplementary material for: The Free Energy Landscape of Small Molecule Unbinding
Source: PLoS Comput Biol. 2011 Feb 3;7(2):e1002002. doi: 10.1371/journal.pcbi.1002002 (PMC3033371; doi:10.1371/journal.pcbi.1002002)
Supplement: Text S1 — This file contains the supporting figures and table for this article. Figure S1: Time series of DRMS from the X-ray structure for 20 of the 50 runs of BUT at 310 K. The y axis is DRMS in Å and x axis is time in ns. Figure S2: Time series of distance between centers of mass of BUT and FKBP active site in 20 of the 50 runs at 310 K. The y axis is distance in Å and x axis is time in ns. The green or red line indicates distance at 15 or 10 Å. Figure S3: Scatter plot of experimental binding energies versus natural logarithm of the unbinding times extracted from MD at 310 and 350 K. The Pearson correlation coefficient is −0.84 and −0.83 for 310 and 350 K MD runs, respectively. The unbinding time and error for each ligand are evaluated by single-exponential fitting of the cumulative distribution function of unbinding times using 25 randomly selected MD runs out of 50, and calculating the average error for the remaining 25 MD runs not used for the fitting, i.e., the difference between the value predicted by the fitting curve and the unbinding time measured along the MD trajectory. This procedure is repeated 100 times for each ligand, and average values of unbinding time and cross-validated error are shown. Figure S4: Cut-based FEPs of six ligands at 310 K (black). The distance between centers of mass of ligand and FKBP active site (green) and the mean first passage time (red) are also shown with y-axis on the right. Figure S5: Network representation of the bound states of the six ligands at 310 K. The largest 25 nodes are marked with numbers and their representatives are shown in Fig. S6–S11 in Text S1. Figure S6: Representative poses of the largest 25 nodes of DMSO. Figure S7: Representative poses of the largest 25 nodes of PENT. Figure S8: Representative poses of the largest 25 nodes of BUT. Figure S9: Representative poses of the largest 25 nodes of DAP. Figure S10: Representative poses of the largest 25 nodes of DSS. Figure S11: Representative poses of the largest 25 nod [file pcbi.1002002.s001.pdf]

Supporting Information

The free energy landscape of small molecule unbinding

Danzhi Huang and Amedeo Caflisch*

[a] Department of Biochemistry
University of Zürich, Winterthurerstrasse 190
CH-8057 Zürich, Switzerland
Phone: (+41 44) 635 55 68, FAX: (+41 44) 635 68 62
email: caflisch@bioc.uzh.ch

* Corresponding author

keywords: FKBP; molecular dynamics; free energy surface; transition state; single exponential kinetics; complex network; Hammond behavior

September 24, 2010

Contents

1 MD simulation of spontaneous unbinding **S-6**

2 Multiple binding modes **S-9**

2.1 MD simulations at 310 K S-9

2.2 Determination of subbasins within the bound state of BUT S-17

2.3 Simplified network of BUT S-18

2.4 MD simulations at 350 K S-19

3 Single-exponential kinetics of unbinding **S-21**

4 Multiple unbinding pathways for 6 ligands **S-23**

5 Robustness with respect to choice of starting pose **S-25**

6 Robustness with respect to choice of DRMS cutoff used for clustering **S-27**

7 Unbinding transition state and Hammond effect **S-28**

8 Diffusivity test **S-29**

9 References **S-30**

List of Figures

S1 Time series of DRMS from the X-ray structure for 20 of the 50 runs of BUT at 310 K. The y axis is DRMS in Å and x axis is time in ns. S-6

S2 Time series of distance between centers of mass of BUT and FKBP active site in 20 of the 50 runs at 310 K. The y axis is distance in Å and x axis is time in ns. The green or red line indicates distance at 15 or 10 Å S-7

S3 Scatter plot of experimental binding energies versus natural logarithm of the unbinding times extracted from MD at 310 and 350 K. The Pearson correlation coefficient is -0.84 and -0.83 for 310 and 350 K MD runs, respectively. The unbinding time and error for each ligand are evaluated by single-exponential fitting of the cumulative distribution function of unbinding times using 25 randomly selected MD runs out of 50, and calculating the average error for the remaining 25 MD runs not used for the fitting, i.e., the difference between the value predicted by the fitting curve and the unbinding time measured along the MD trajectory. This procedure is repeated 100 times for each ligand, and average values of unbinding time and cross-validated error are shown. S-8

S4 Cut-based FEPs of six ligands at 310 K (black). The distance between centers of mass of ligand and FKBP active site (green) and the mean first passage time (red) are also shown with y-axis on the right. S-9

S5 Network representation of the bound states of the six ligands at 310 K. The largest 25 nodes are marked with numbers and their representatives are shown in Fig. S6-S11. . . . . S-10

S6 Representative poses of the largest 25 nodes of DMSO. S-11

S7 Representative poses of the largest 25 nodes of PENT. S-12

S8 Representative poses of the largest 25 nodes of BUT. S-13

S9 Representative poses of the largest 25 nodes of DAP. S-14

S10 Representative poses of the largest 25 nodes of DSS. S-15

S11 Representative poses of the largest 25 nodes of THI. S-16

S12 Cut-based FEPs plotted using as reference node the most populated node of individual subbasins. These cut-based FEPs were used to determine the subbasins of the bound state. The cut-based FEP on the top left corresponds to the one in Figure 2 of the main text. . . S-17

S13 Simplified network of subbasins in the bound state of BUT. The nodes are the subbasins identified with the procedure shown in Figure S12 except for the black node which represents the unbound state. The thickness of the links is proportional to the number of the transitions observed in the 50 MD runs at 310 K. S-18

S14 Network representation of the bound states of the six ligands at 350 K. Only nodes connected by links of weight 5 or more are shown to avoid overcrowding. S-19

S15 Cut-based FEPs of six ligands at 350 K. S-20

S16 Single-exponential kinetics of unbinding for 6 ligands at 310 K. The plots show the cumulative distribution f(t) of the unbinding times observed in the 50 MD runs. Note that the unbinding times obtained by fitting are slightly different from those in Table 1 of the main text because a cross-validation procedure was used in the latter. S-21

S17 Single-exponential kinetics of unbinding for 6 ligands at 350 K. The plots show the cumulative distribution f(t) of the unbinding times for 6 ligands at 350 K. The unbinding times range from 1.6 to 5.6 ns, which is shorter than the corresponding values at 310 K. S-22

S18 Network representations of the bound state for DMSO (top left), PENT (top right), BUT (middle left), DAP (middle right), DSS (bottom left), and THI (bottom right). Nodes are colored from red to green according to the distance of the centers of mass of ligand and FKBP. S-23

S19 Stereoview of the most populated clusters for 6 ligands - DMSO, PENT, BUT, DAP, DSS and THI (top to bottom). Nodes are colored from red to green according to the distance of the centers of mass of ligand and FKBP. S-24

S20 Test at 310 K with DMSO. Ten bound state conformations were randomly chosen from previous MD simulations and ten runs of 10 ns each with different initial velocities were started for each of them. Single-exponential kinetics of unbinding is observed and the unbinding time derived from the plot is 4.2 ns which is similar to the value derived from the 50 runs started from the X-ray structure of the complex. S-25

S21 Test at 350 K with DMSO. Fifty 5-ns runs with different velocities were started for each of five randomly oriented poses of DMSO in the active site of FKBP. Single-exponential kinetics of unbinding is observed and the unbinding times derived from the plots range from 1.3 to 1.9 ns, which is consistent with the value derived from the 50 runs started from the X-ray structure of the complex (top,left). . . S-26

S22 The cFEPs for DMSO (left) and PENT (right) were obtained using DRMS clustering cutoffs of 0.8 Å, 0.9 Å, 1.0 Å, and 1.5 Å from top to bottom. S-27

S23 Diffusivity test for the clustering of DMSO and THI. The profiles with saving frequency at 4 and 8 ps are similar upon a vertical shift of $\ln(\sqrt{2})$, which is consistent with the diffusive regime [1]. S-29

List of Tables

S-1 Robustness of TSE definition and Hammond behavior. Each column lists the average distances between the centers of mass of the ligand and FKBP active site for the conformations at the TSE. The numbers of TSE nodes and snapshots are shown in parentheses. Only TSE nodes with weight larger than 5 were used for this analysis as nodes with very low weight increase the noise [2]. S-28

1 MD simulation of spontaneous unbinding

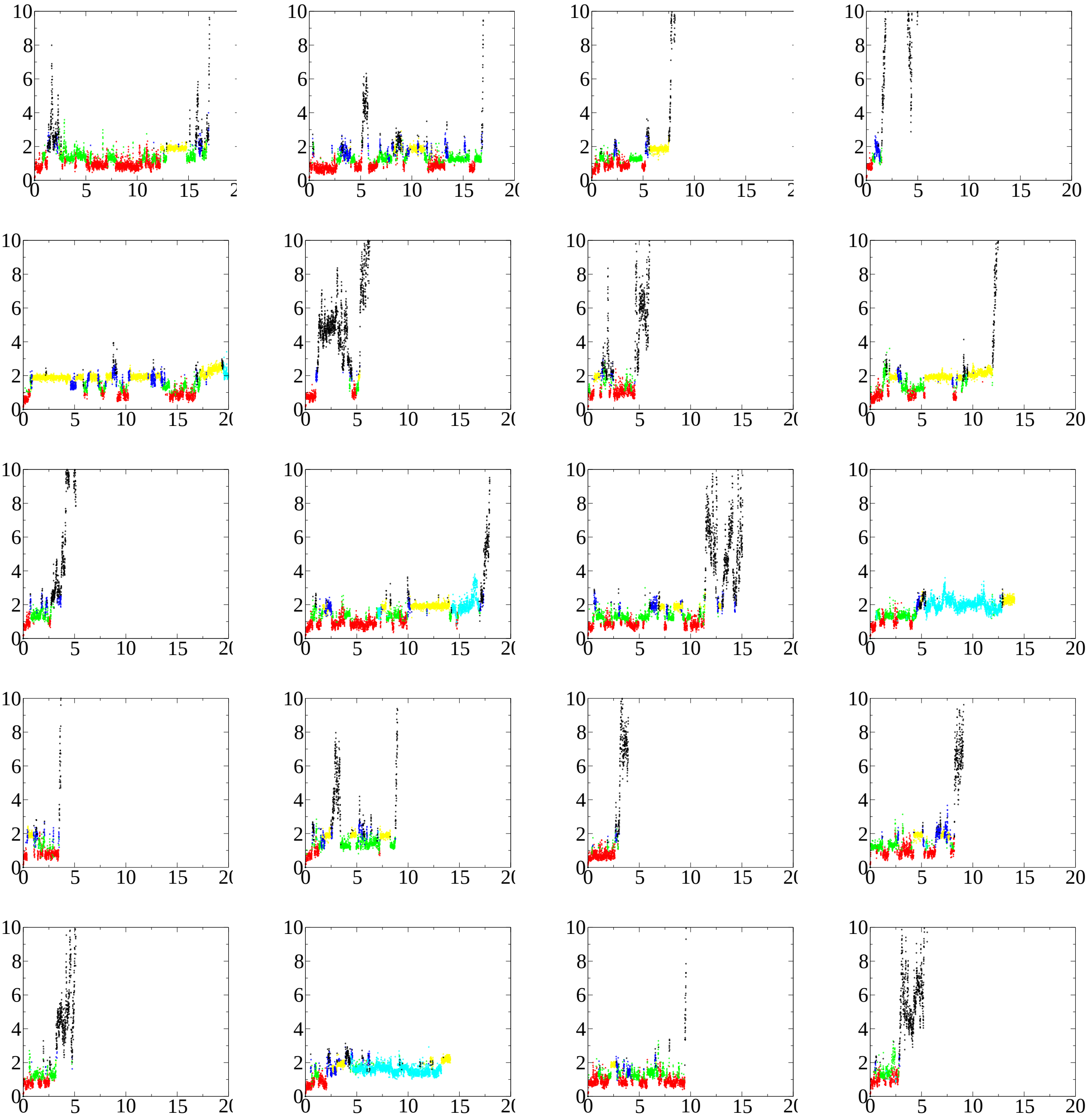

Figure S1: Time series of DRMS from the X-ray structure for 20 of the 50 runs of BUT at 310 K. The y axis is DRMS in Å and x axis is time in ns.

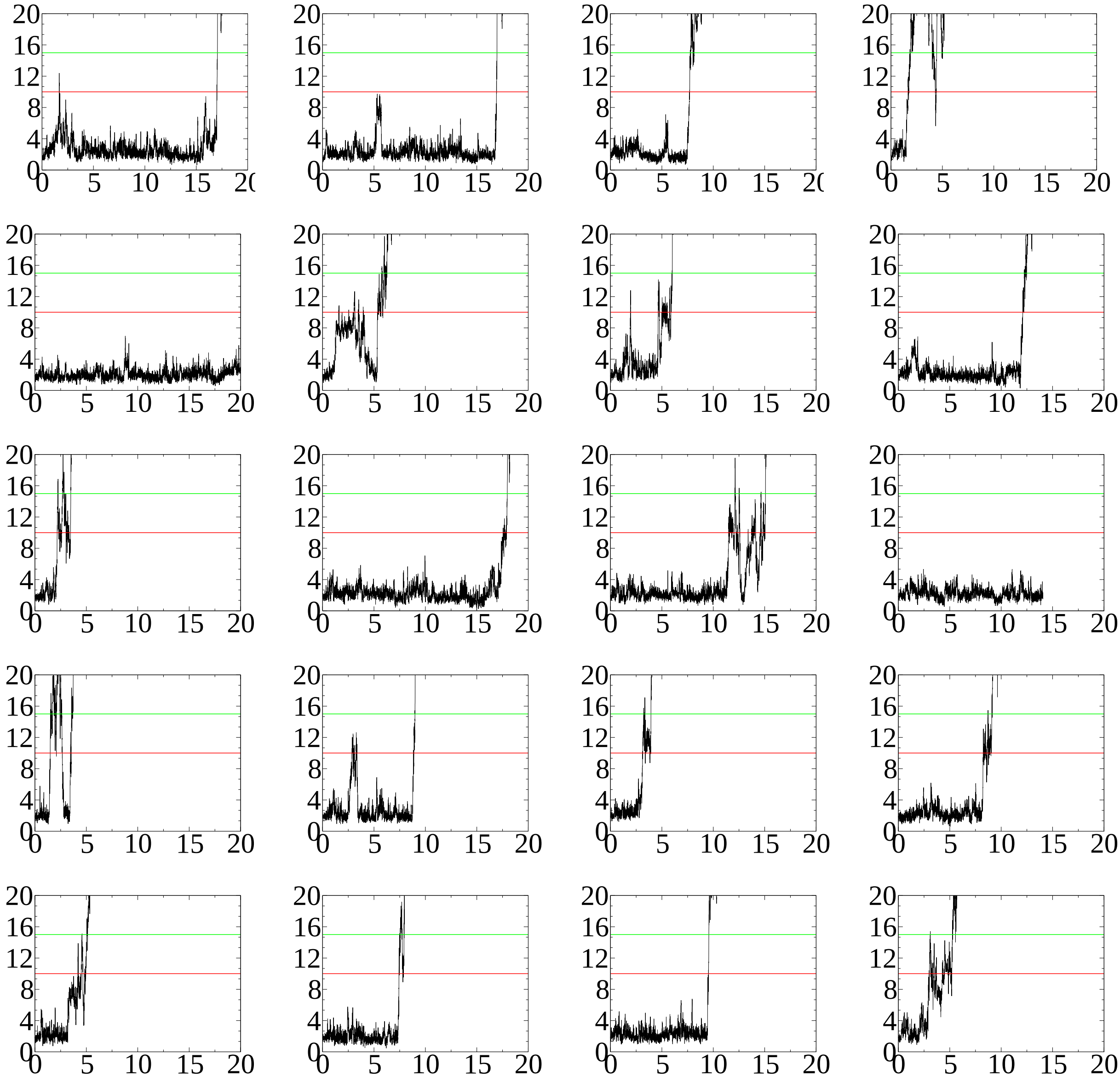

Figure S2: Time series of distance between centers of mass of BUT and FKBP active site in 20 of the 50 runs at 310 K. The y axis is distance in Å and x axis is time in ns. The green or red line indicates distance at 15 or 10 Å .

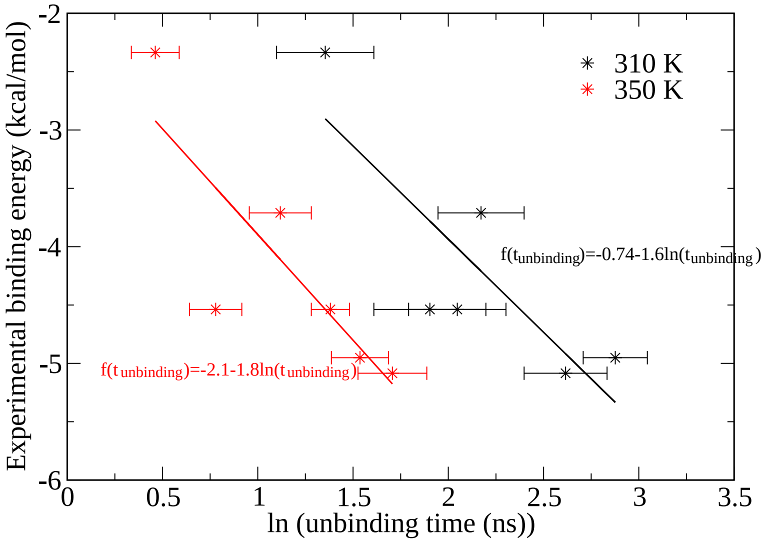

Figure S3: Scatter plot of experimental binding energies versus natural logarithm of the unbinding times extracted from MD at 310 and 350 K. The Pearson correlation coefficient is -0.84 and -0.83 for 310 and 350 K MD runs, respectively. The unbinding time and error for each ligand are evaluated by single-exponential fitting of the cumulative distribution function of unbinding times using 25 randomly selected MD runs out of 50, and calculating the average error for the remaining 25 MD runs not used for the fitting, i.e., the difference between the value predicted by the fitting curve and the unbinding time measured along the MD trajectory. This procedure is repeated 100 times for each ligand, and average values of unbinding time and cross-validated error are shown.

2 Multiple binding modes

2.1 MD simulations at 310 K

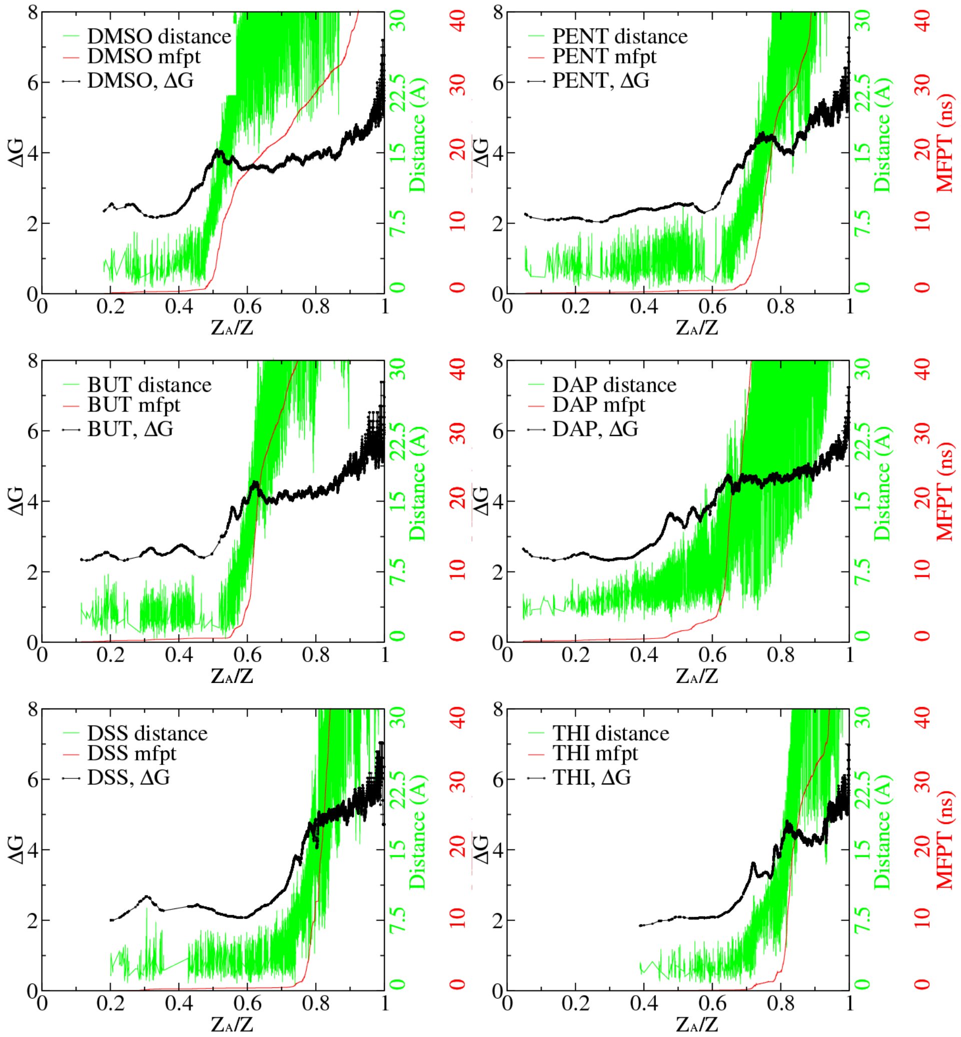

Figure S4: Cut-based FEPs of six ligands at 310 K (black). The distance between centers of mass of ligand and FKBP active site (green) and the mean first passage time (red) are also shown with y-axis on the right.

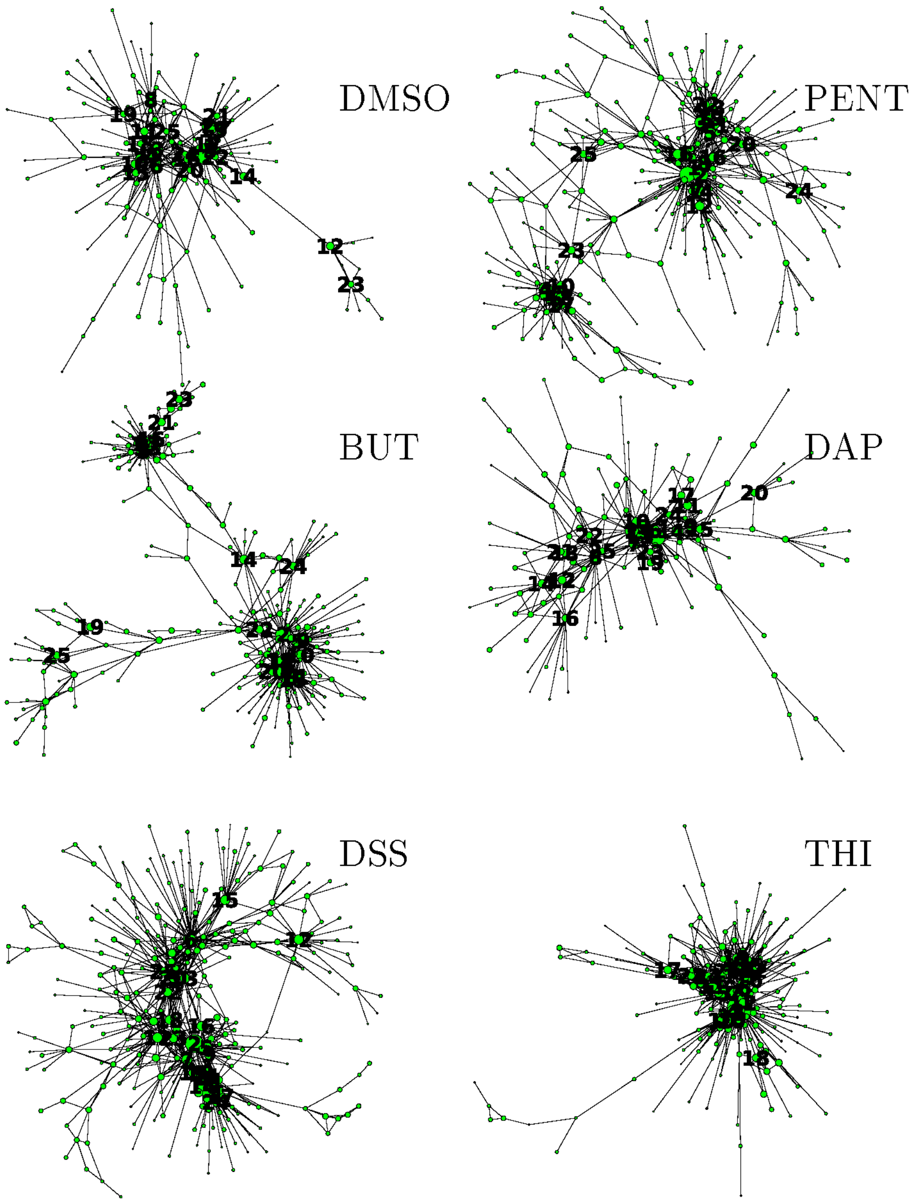

Figure S5: Network representation of the bound states of the six ligands at 310 K. The largest 25 nodes are marked with numbers and their representatives are shown in Fig. S6-S11.

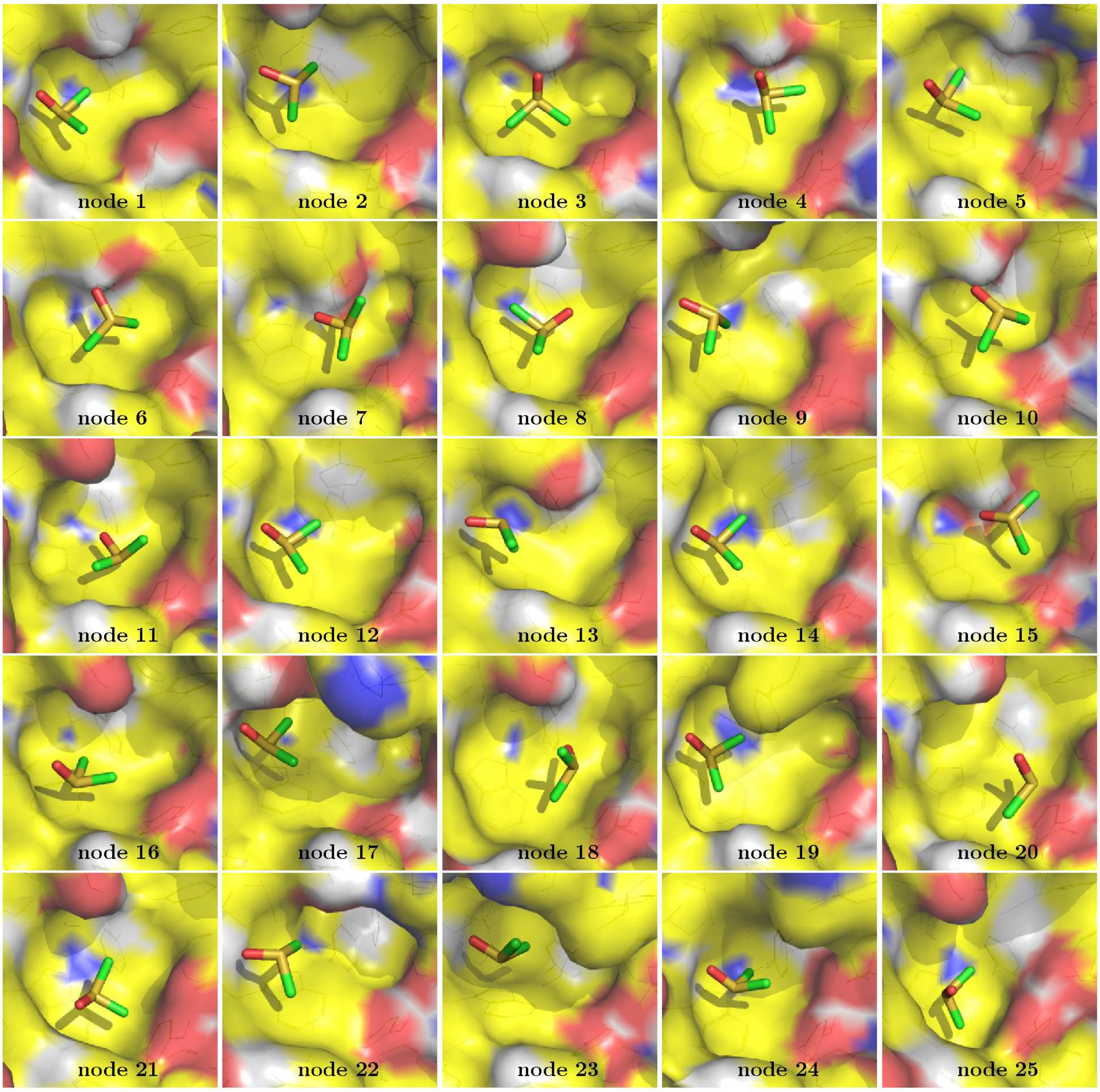

Figure S6: Representative poses of the largest 25 nodes of DMSO.

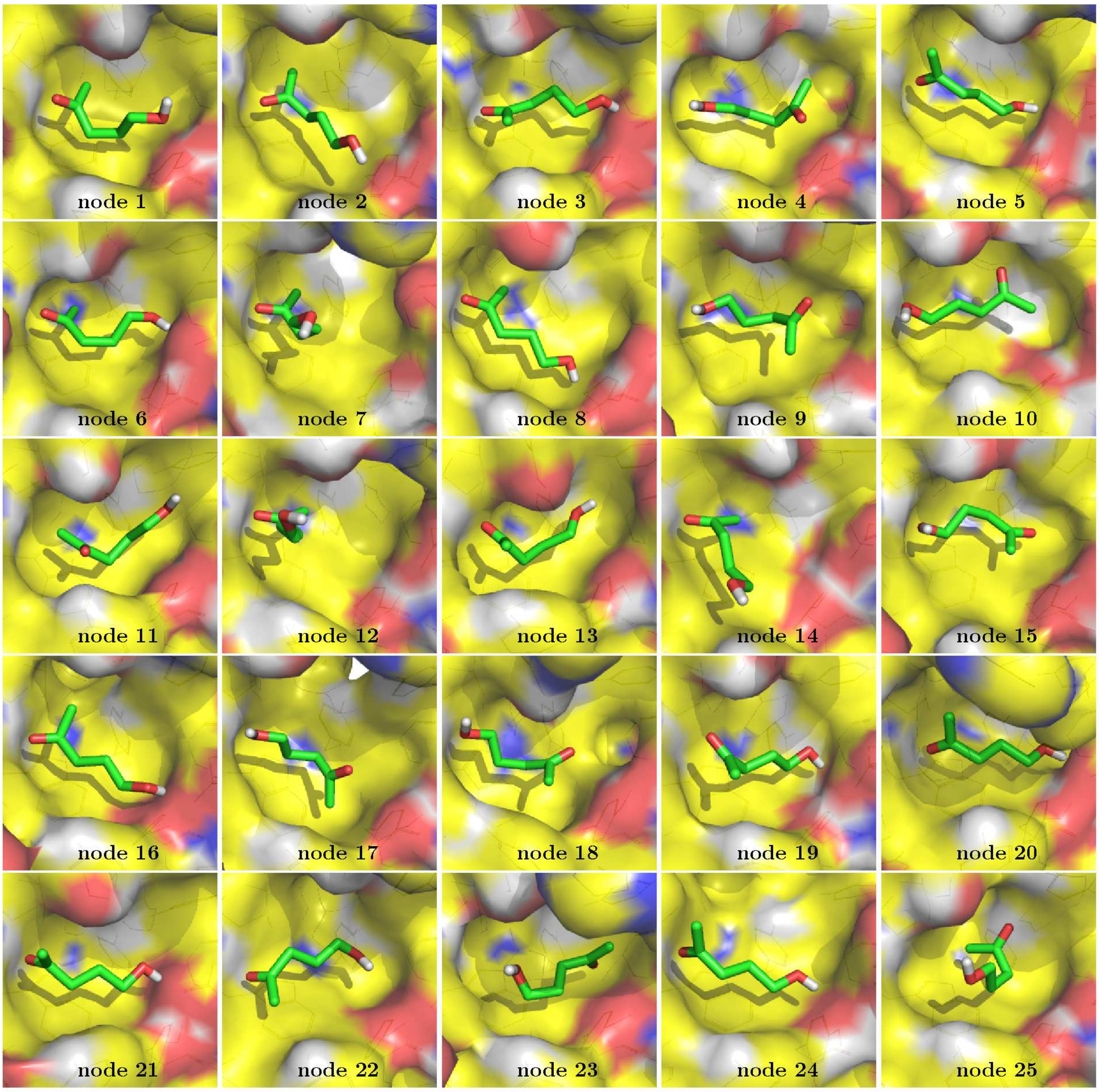

Figure S7: Representative poses of the largest 25 nodes of PENT.

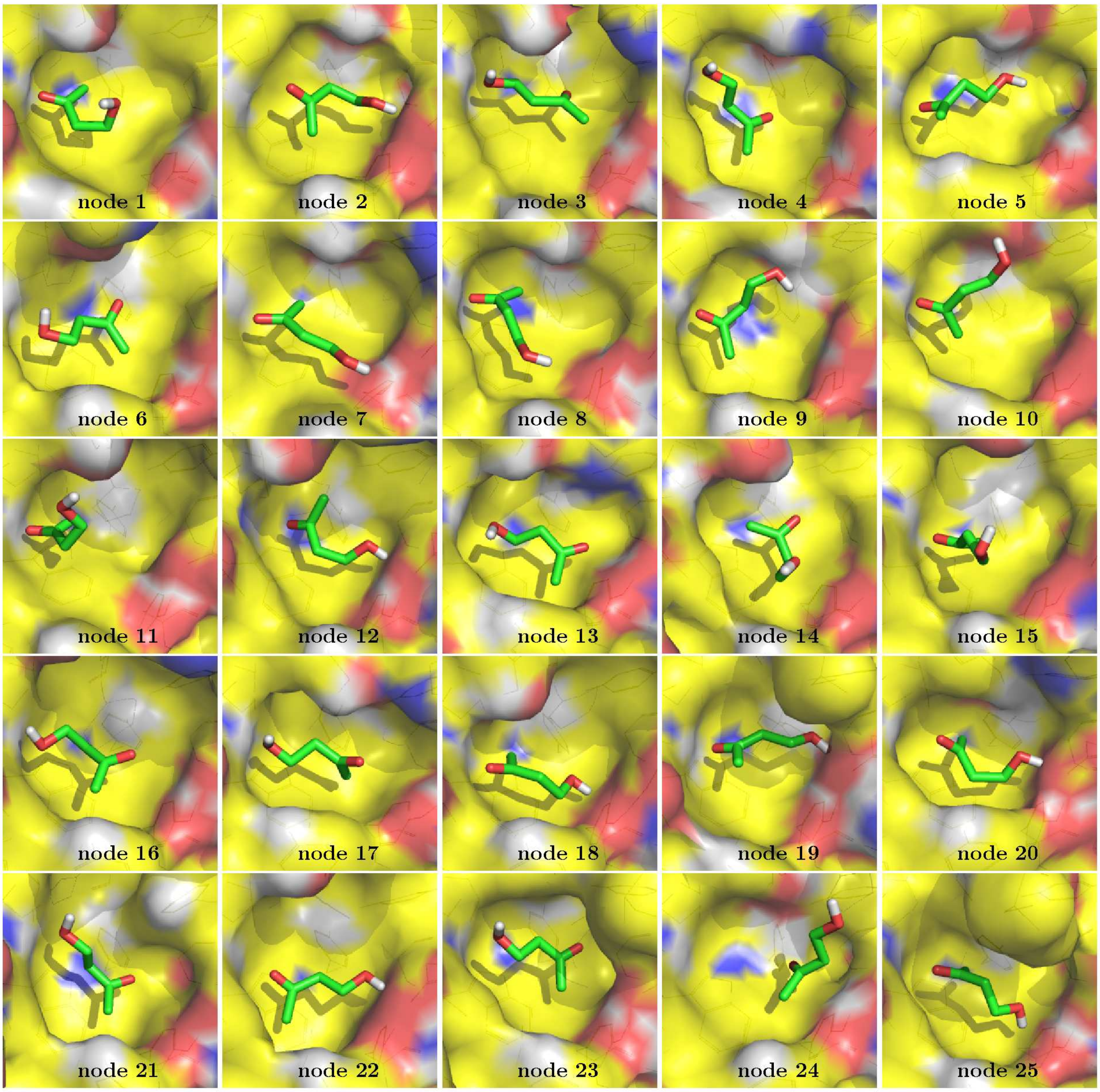

Figure S8: Representative poses of the largest 25 nodes of BUT.

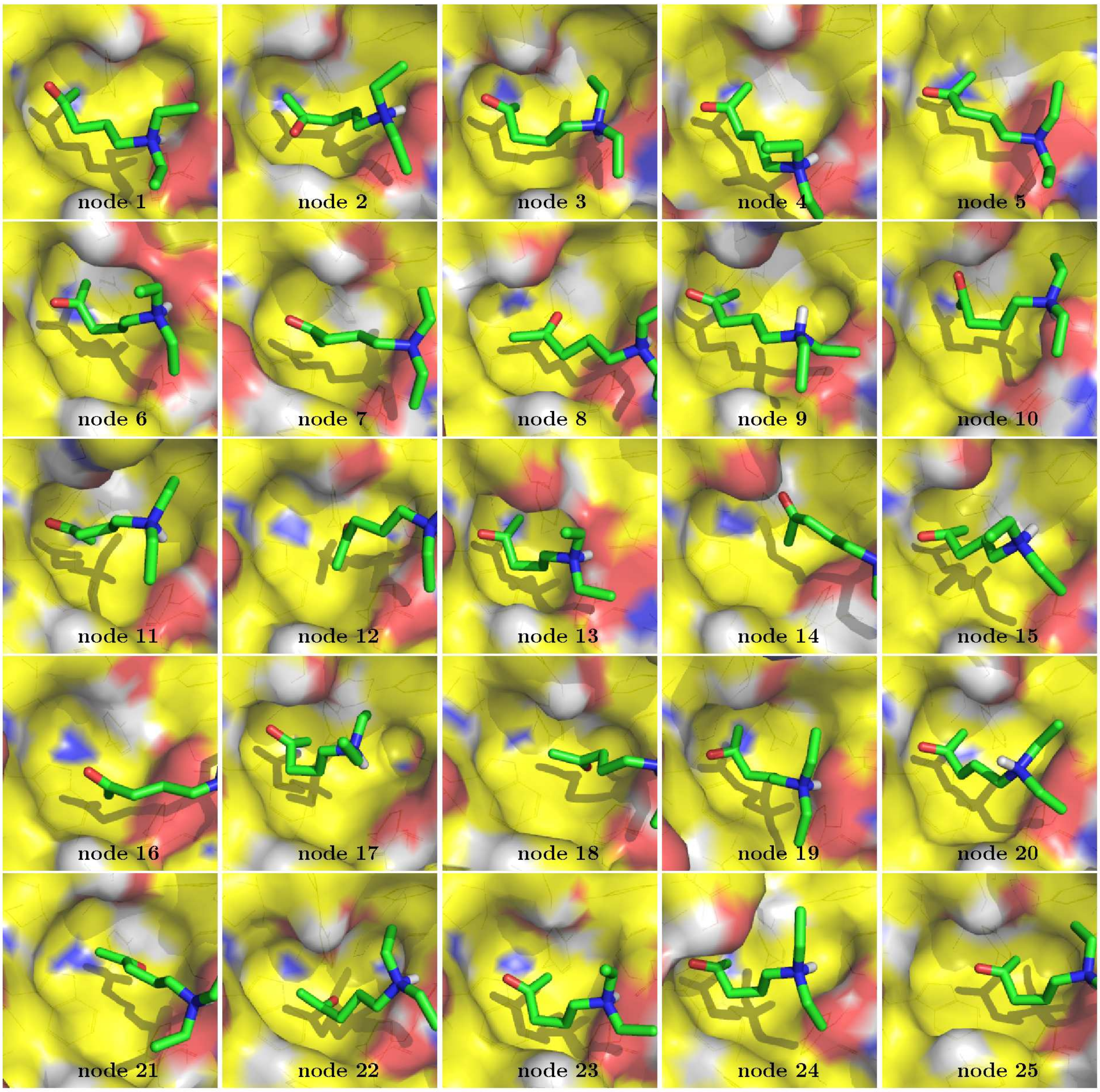

Figure S9: Representative poses of the largest 25 nodes of DAP.

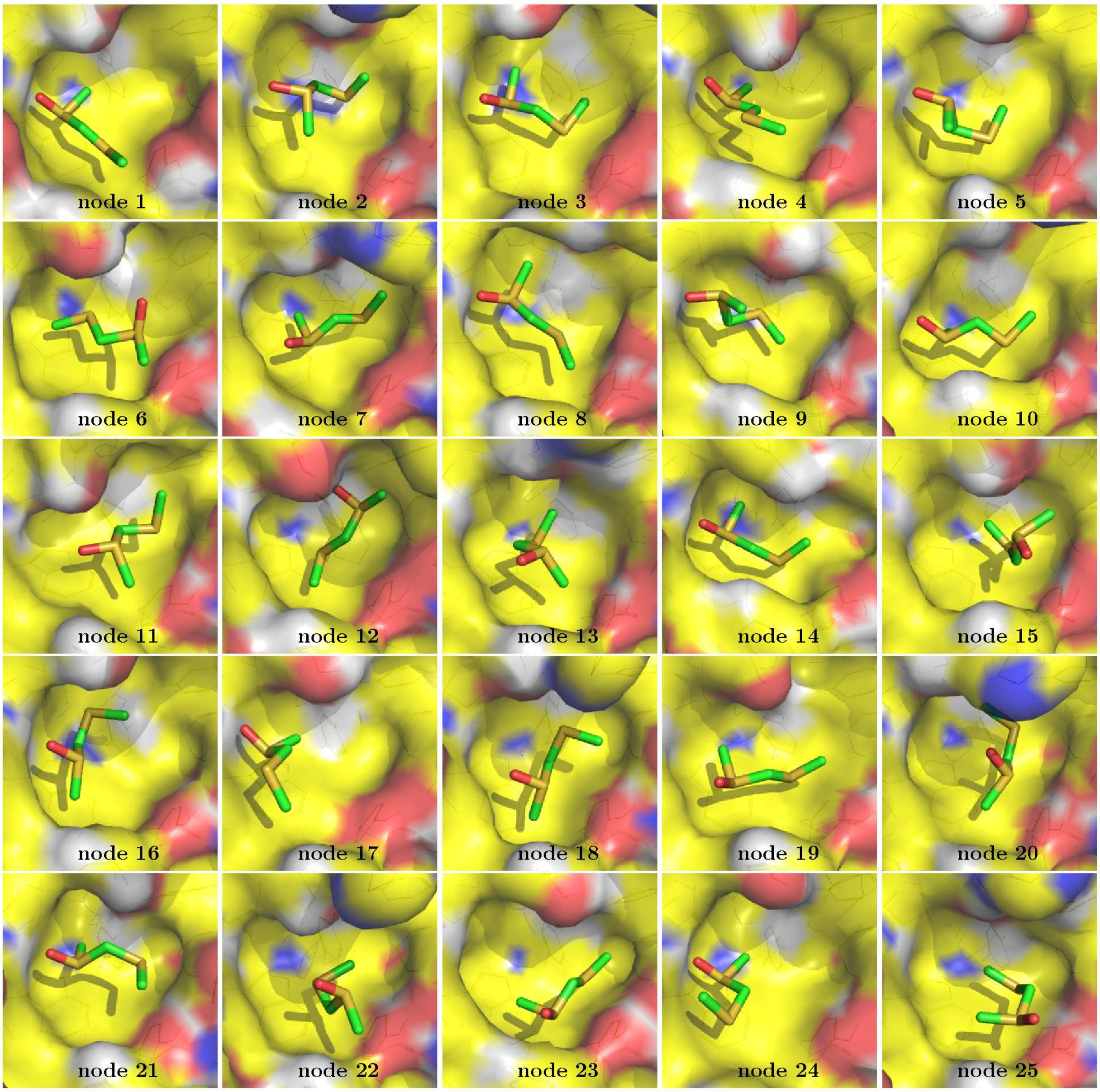

Figure S10: Representative poses of the largest 25 nodes of DSS.

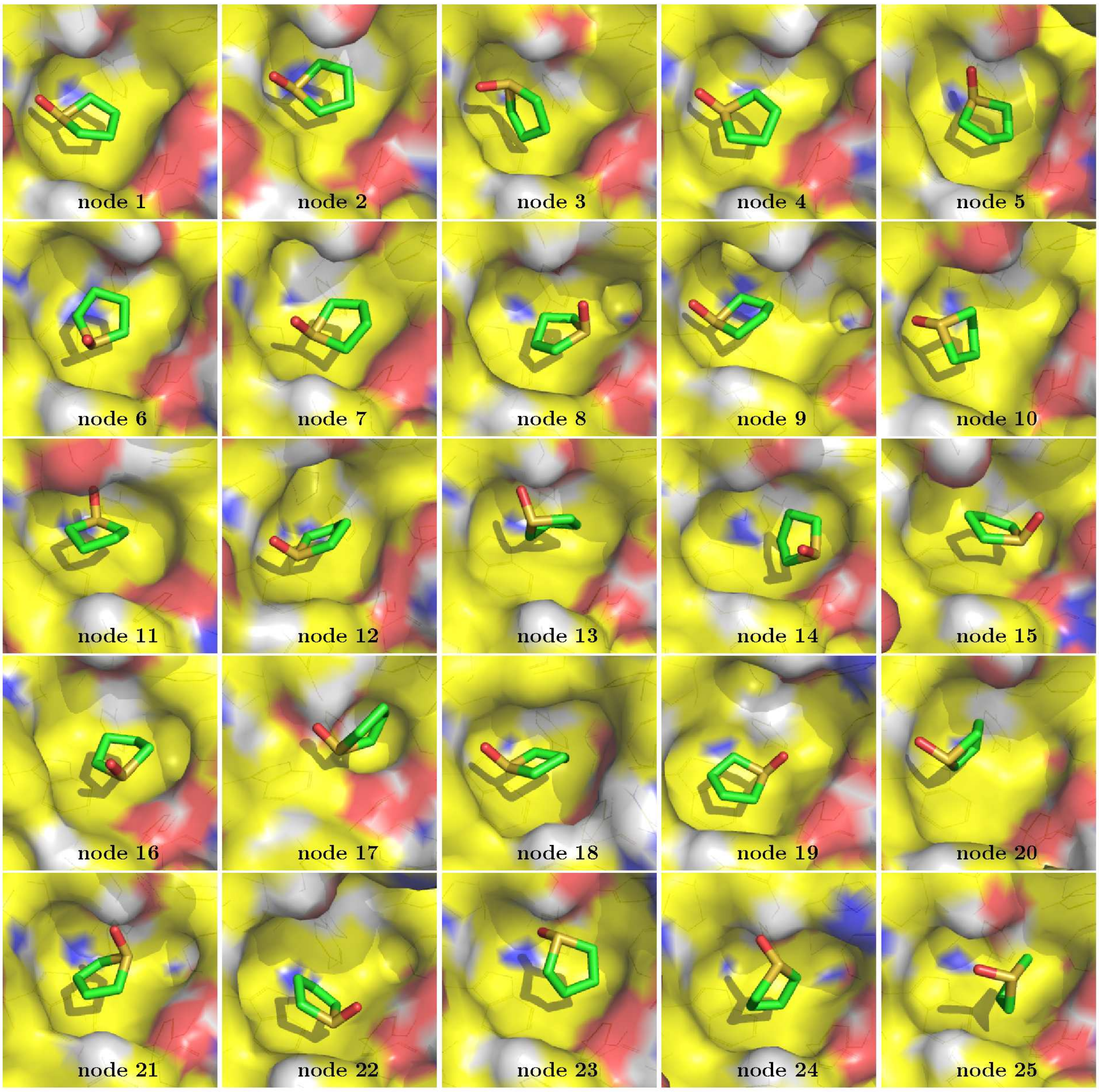

Figure S11: Representative poses of the largest 25 nodes of THI.

2.2 Determination of subbasins within the bound state of BUT

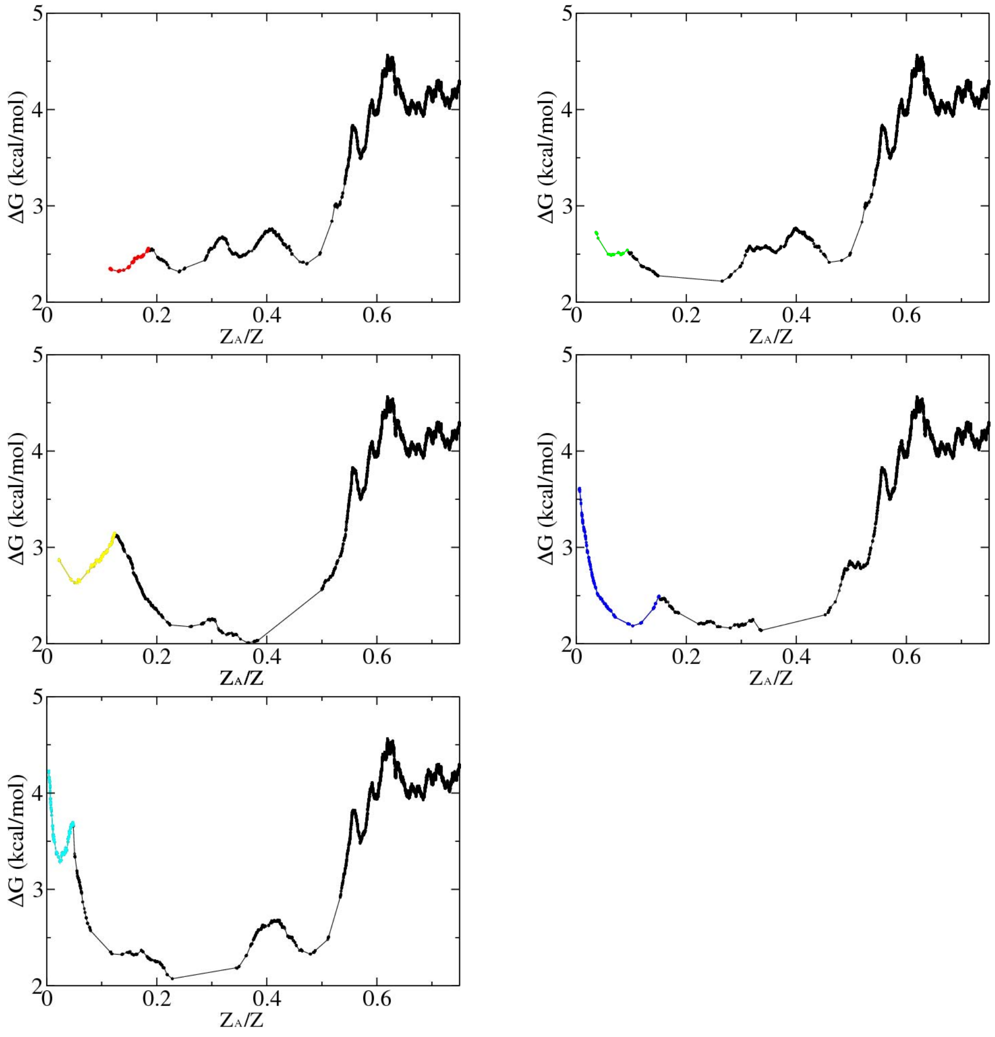

Figure S12: Cut-based FEPs plotted using as reference node the most populated node of individual subbasins. These cut-based FEPs were used to determine the subbasins of the bound state. The cut-based FEP on the top left corresponds to the one in Figure 2 of the main text.

2.3 Simplified network of BUT

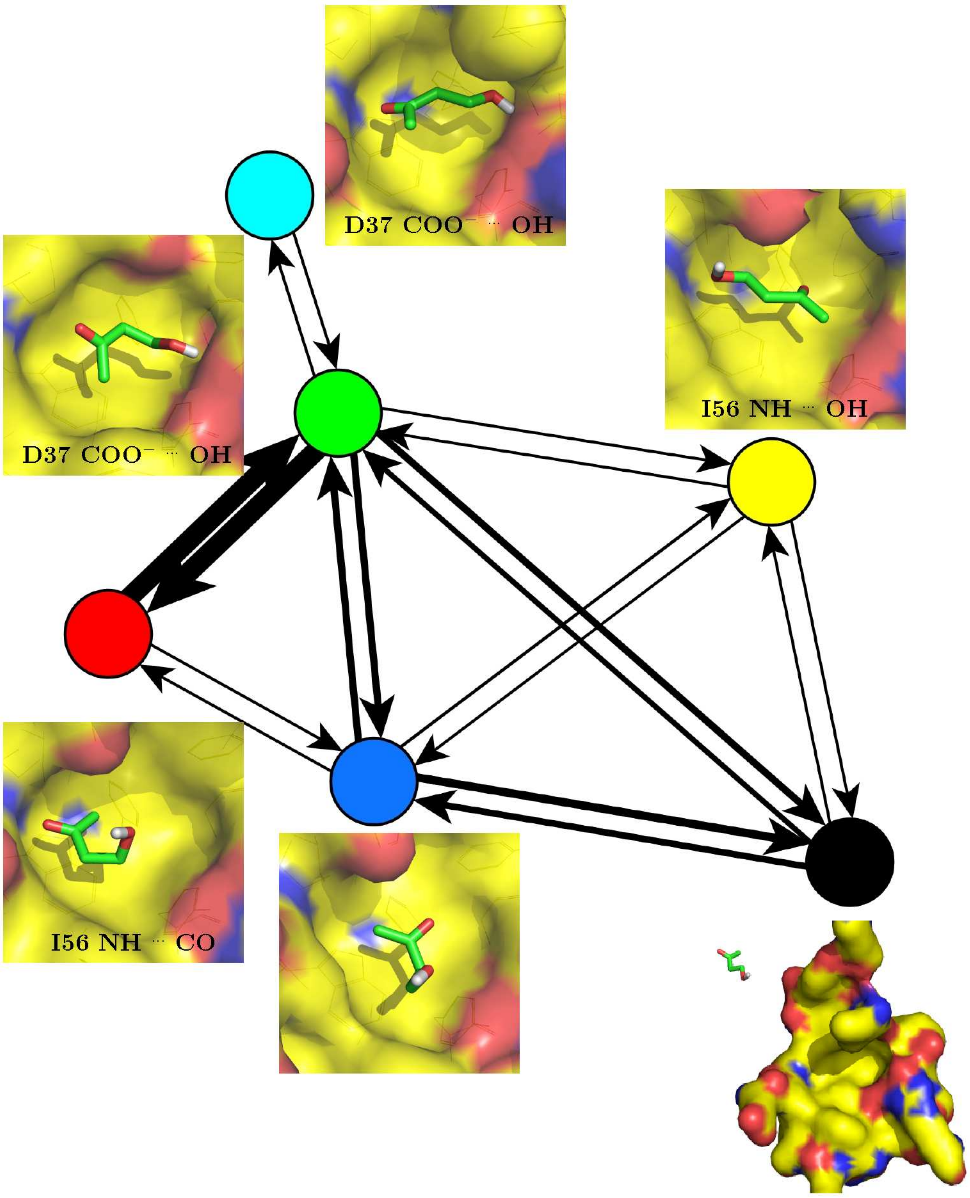

Figure S13: Simplified network of subbasins in the bound state of BUT. The nodes are the subbasins identified with the procedure shown in Figure S12 except for the black node which represents the unbound state. The thickness of the links is proportional to the number of the transitions observed in the 50 MD runs at 310 K.

2.4 MD simulations at 350 K

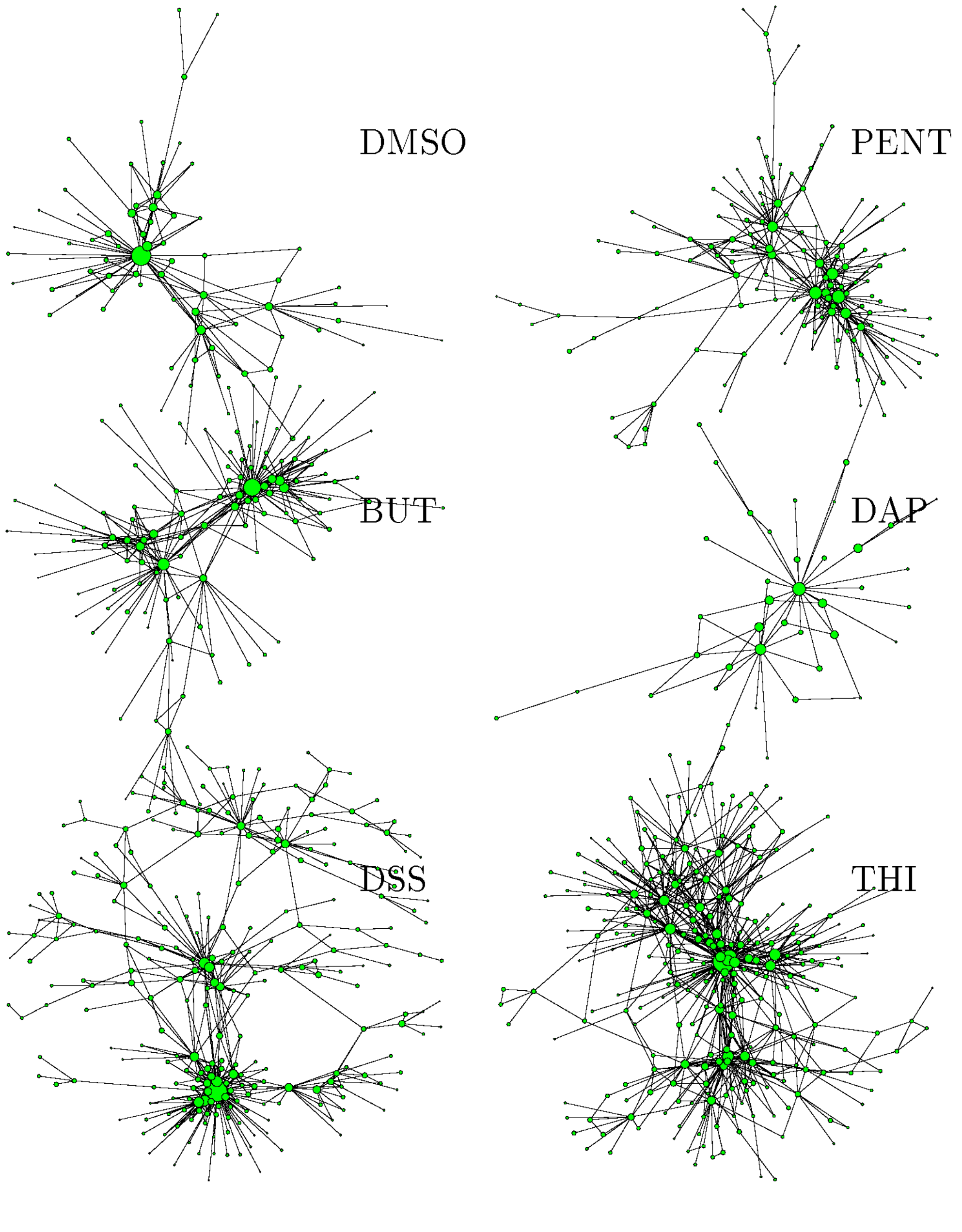

Figure S14: Network representation of the bound states of the six ligands at 350 K. Only nodes connected by links of weight 5 or more are shown to avoid overcrowding.

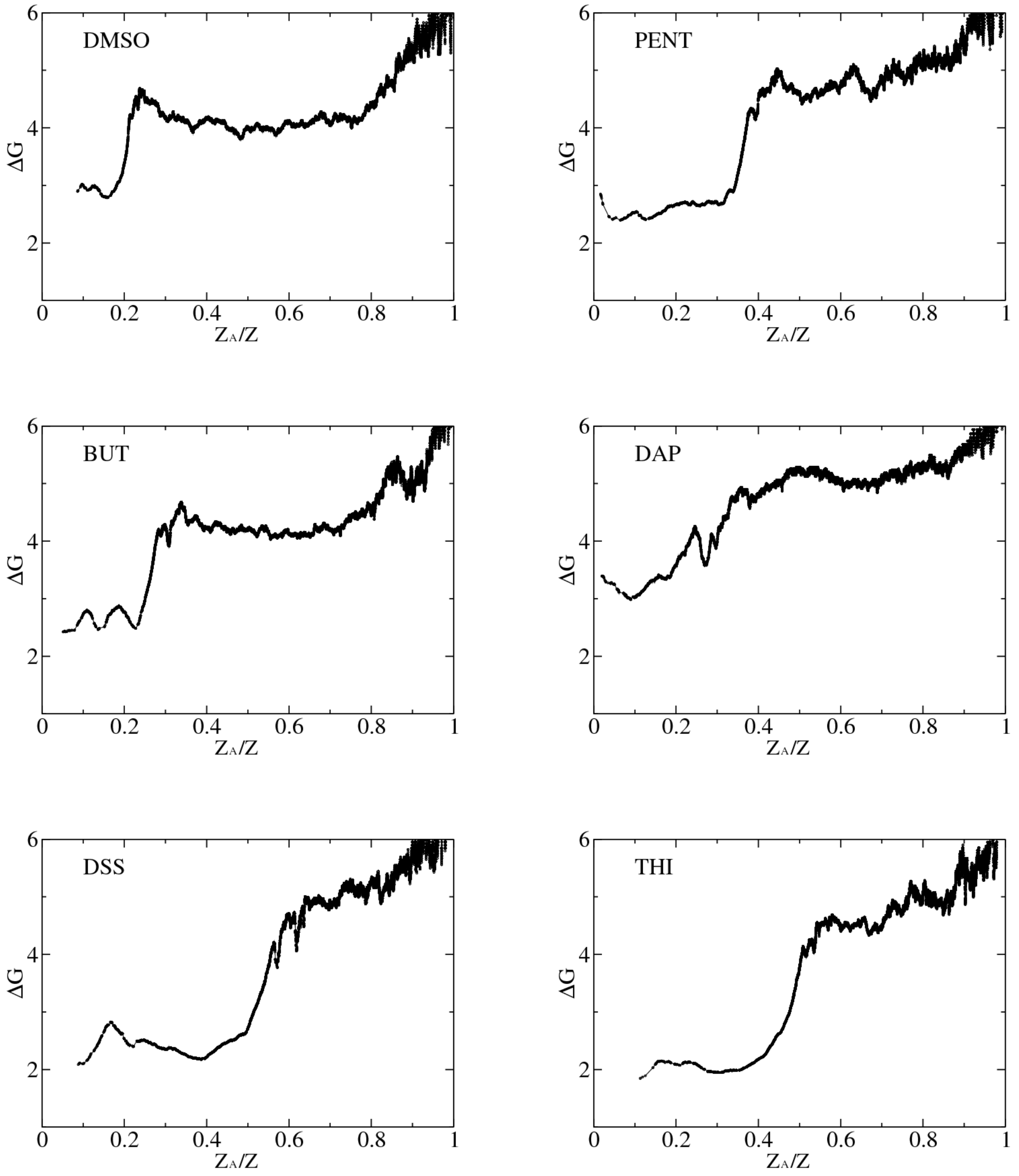

Figure S15: Cut-based FEPs of six ligands at 350 K.

3 Single-exponential kinetics of unbinding

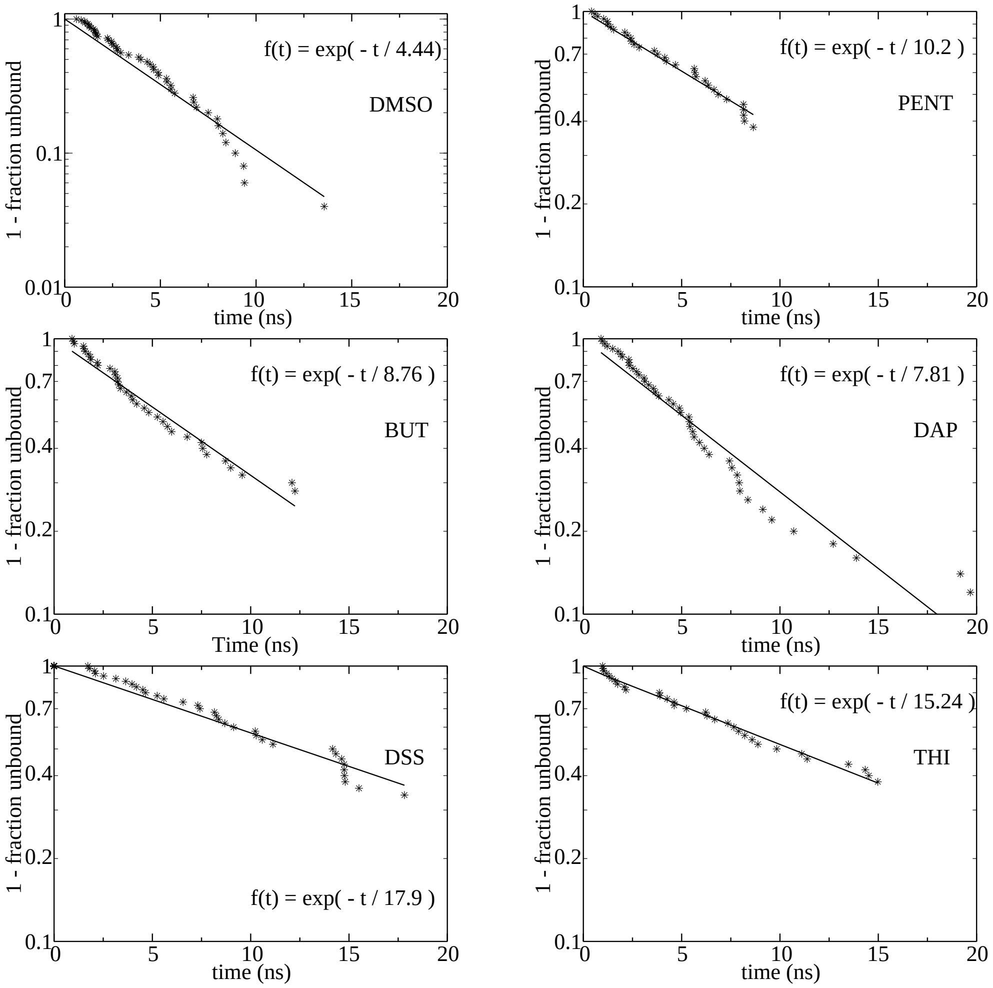

Figure S16: Single-exponential kinetics of unbinding for 6 ligands at 310 K. The plots show the cumulative distribution f(t) of the unbinding times observed in the 50 MD runs. Note that the unbinding times obtained by fitting are slightly different from those in Table 1 of the main text because a cross-validation procedure was used in the latter.

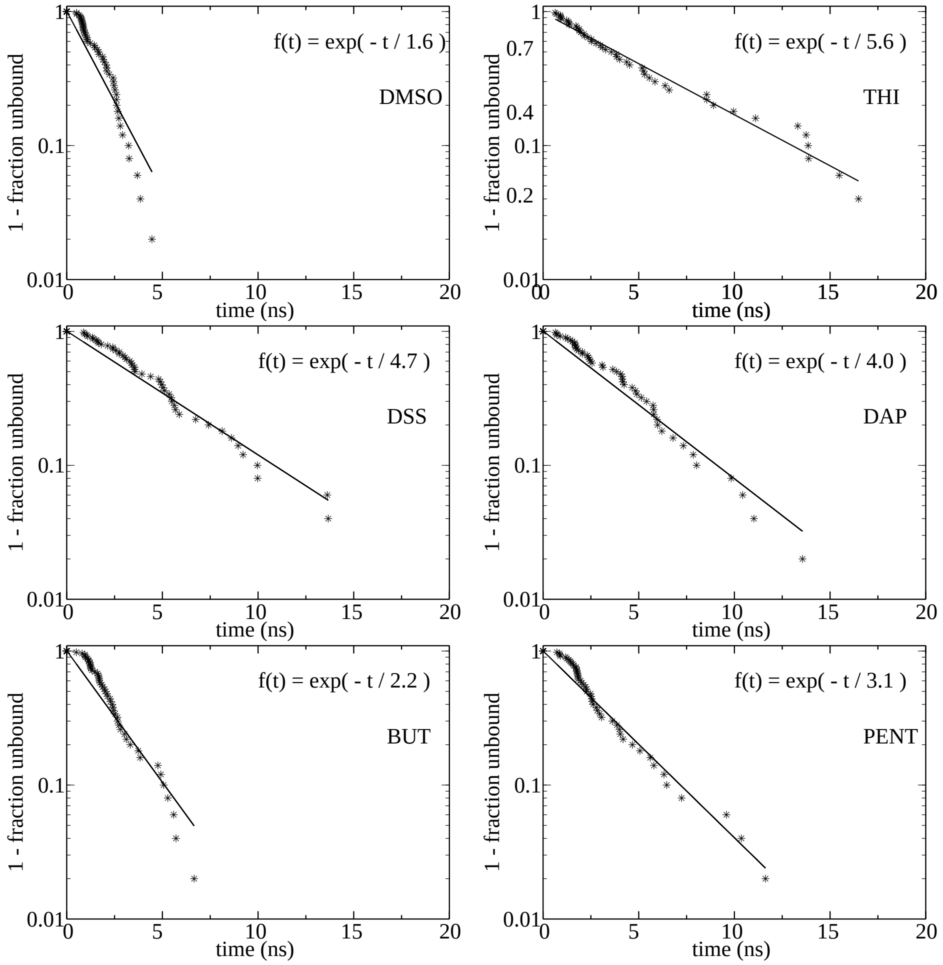

Figure S17: Single-exponential kinetics of unbinding for 6 ligands at 350 K. The plots show the cumulative distribution f(t) of the unbinding times for 6 ligands at 350 K. The unbinding times range from 1.6 to 5.6 ns, which is shorter than the corresponding values at 310 K.

4 Multiple unbinding pathways for 6 ligands

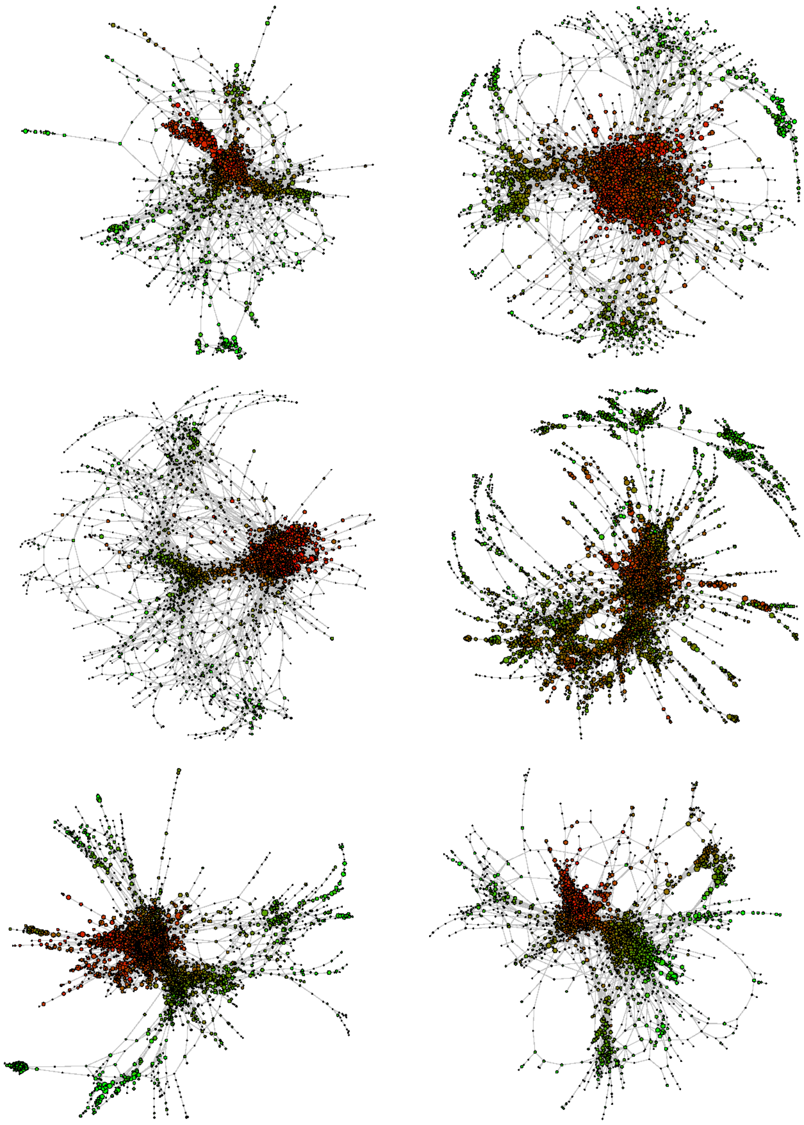

Figure S18: Network representations of the bound state for DMSO (top left), PENT (top right), BUT (middle left), DAP (middle right), DSS (bottom left), and THI (bottom right). Nodes are colored from red to green according to the distance of the centers of mass of ligand and FKBP.

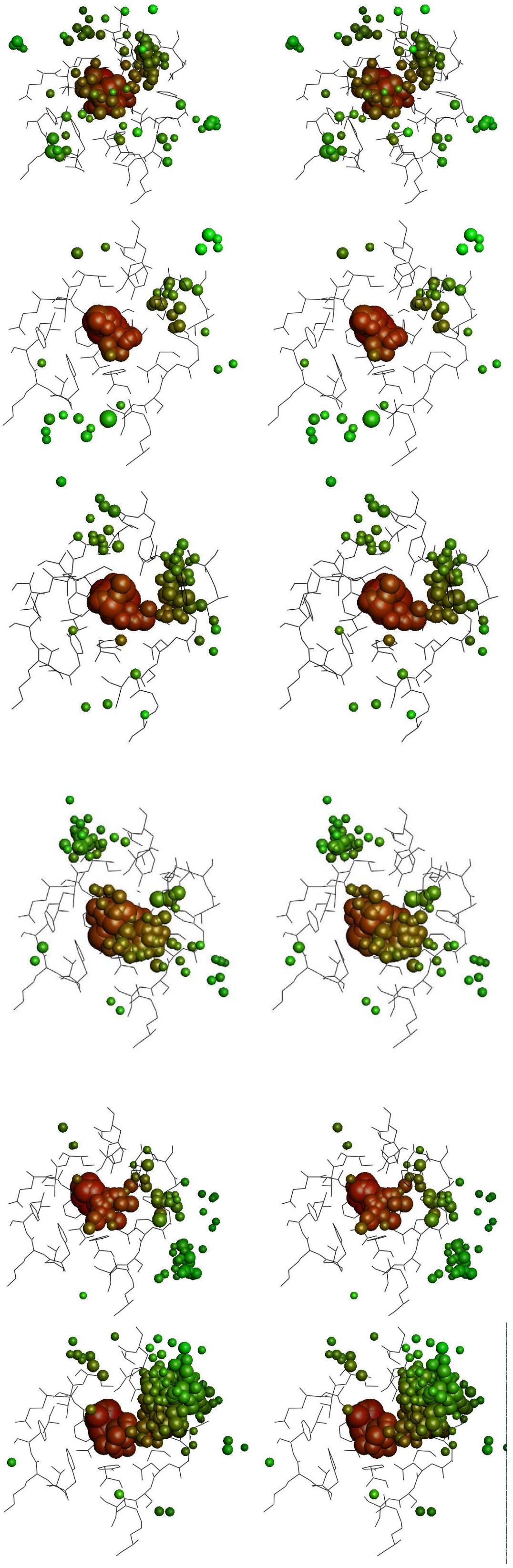

Figure S19: Stereoview of the most populated clusters for 6 ligands - DMSO, PENT, BUT, DAP, DSS and THI (top to bottom). Nodes are colored from red to green according to the distance of the centers of mass of ligand and FKBP.

5 Robustness with respect to choice of starting pose

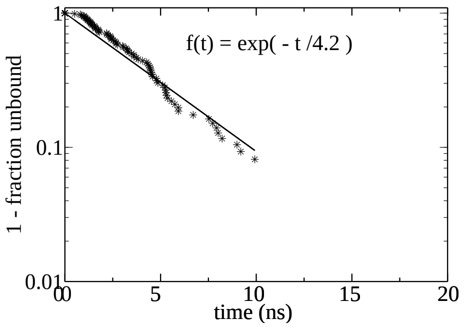

Figure S20: Test at 310 K with DMSO. Ten bound state conformations were randomly chosen from previous MD simulations and ten runs of 10 ns each with different initial velocities were started for each of them. Single-exponential kinetics of unbinding is observed and the unbinding time derived from the plot is 4.2 ns which is similar to the value derived from the 50 runs started from the X-ray structure of the complex.

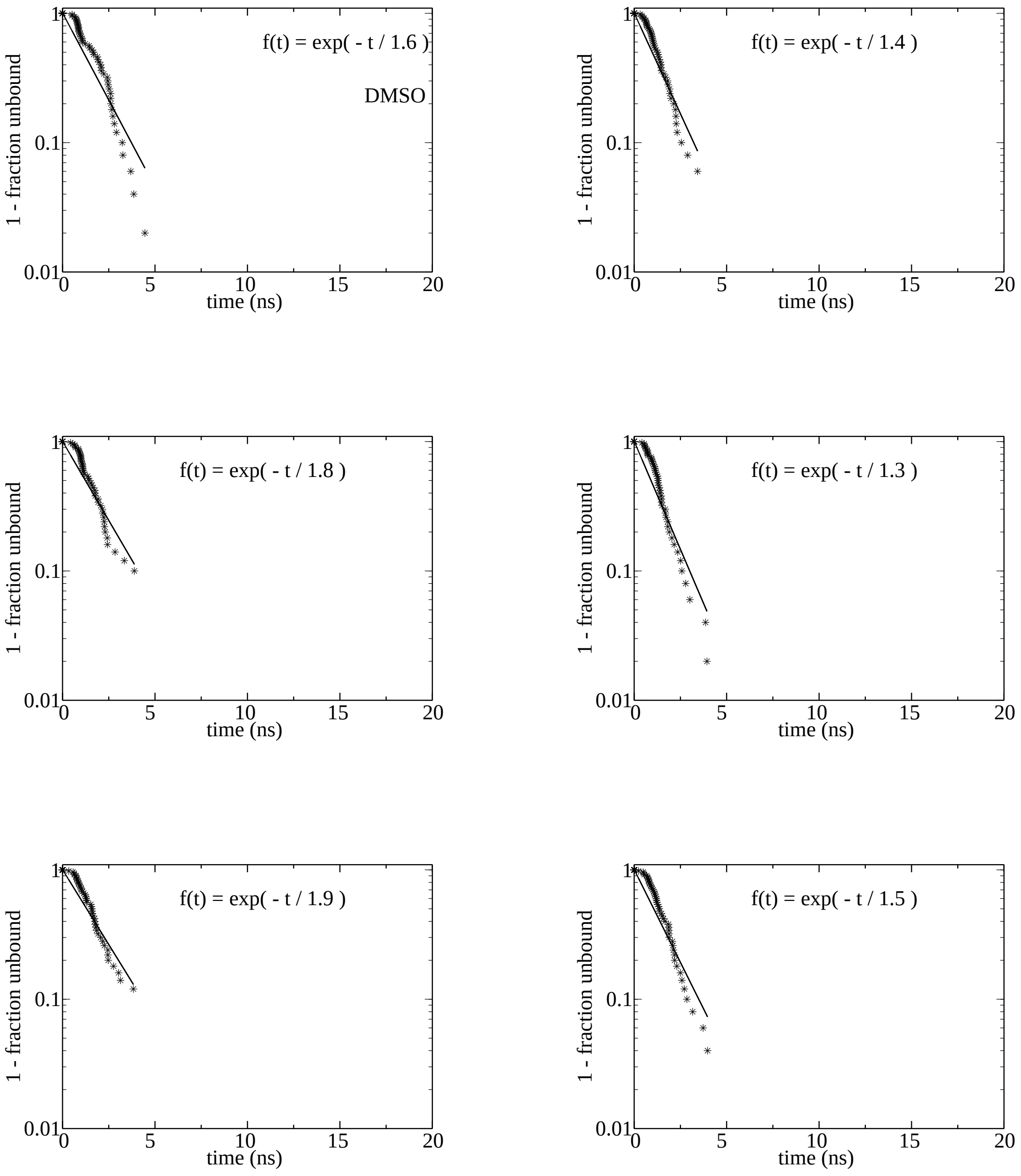

Figure S21: Test at 350 K with DMSO. Fifty 5-ns runs with different velocities were started for each of five randomly oriented poses of DMSO in the active site of FKBP. Single-exponential kinetics of unbinding is observed and the unbinding times derived from the plots range from 1.3 to 1.9 ns, which is consistent with the value derived from the 50 runs started from the X-ray structure of the complex (top,left).

6 Robustness with respect to choice of DRMS cutoff used for clustering

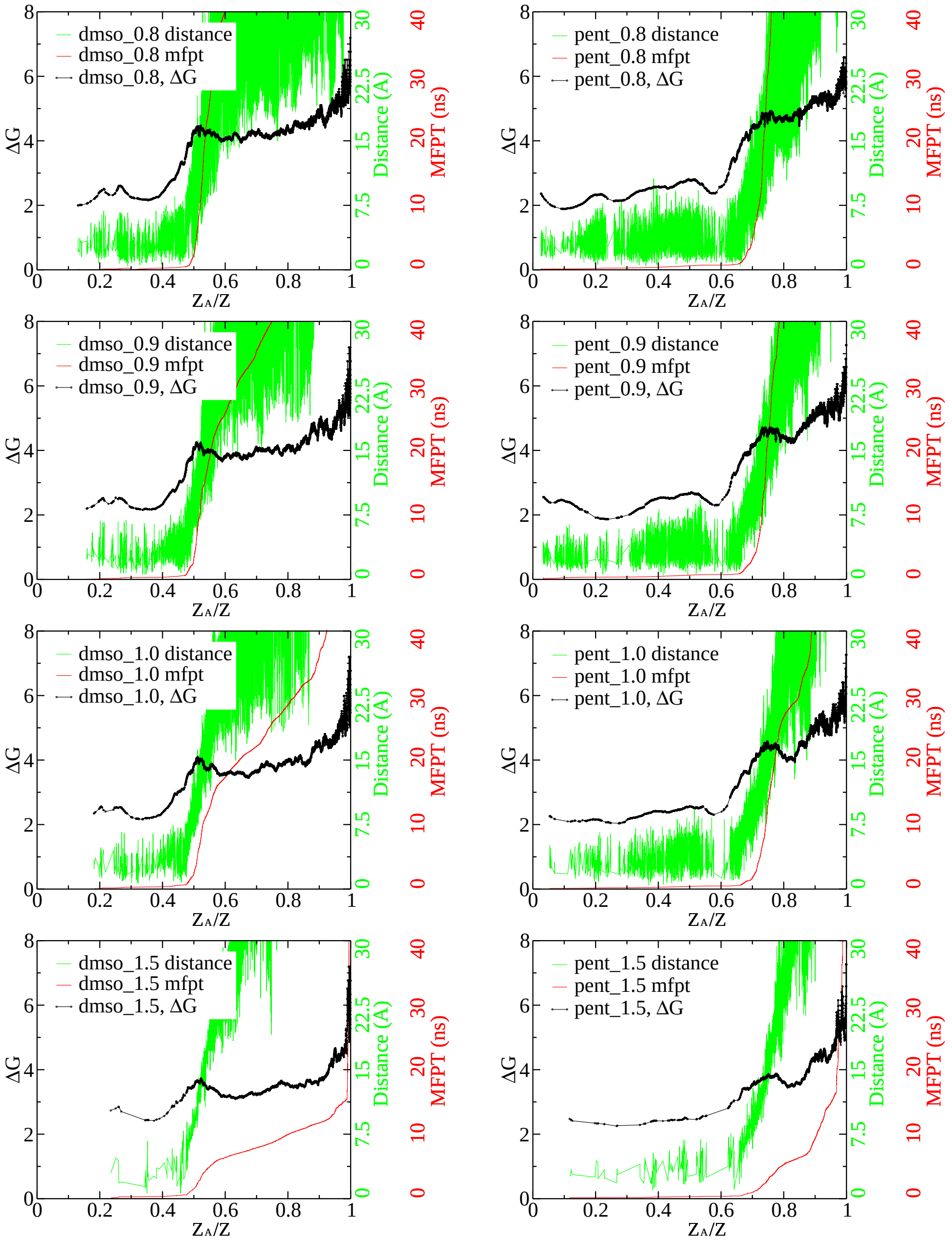

Figure S22: The cFEPs for DMSO (left) and PENT (right) were obtained using DRMS clustering cutoffs of 0.8 Å, 0.9 Å, 1.0 Å, and 1.5 Å from top to bottom.

7 Unbinding transition state and Hammond effect

		Affinity	Average position of TSE along unbinding reaction coordinate (Å)			
		[mM]	$\tau_{commit} = 0.4\ ns$	$\tau_{commit} = 0.4\ ns$	$\tau_{commit} = 0.8\ ns$	$\tau_{commit} = 0.8\ ns$
			$0.45 < P^N_{unbind} < 0.55$	$0.4 < P^N_{unbind} < 0.6$	$0.45 < P^N_{unbind} < 0.55$	$0.4 < P^N_{unbind} < 0.6$
DMSO	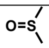	20	5.3 (25, 300)	6.1 (81, 814)	4.7 (40, 852)	4.8 (118, 1847)
PENT	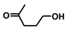	2	6.5 (19, 253)	7.1 (65, 576)	5.4 (38, 619)	5.7 (103, 1195)
BUT	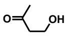	0.5	6.9 (22, 296)	7.4 (77, 691)	5.6 (34, 560)	6.2 (109, 1220)
DAP	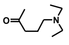	0.5	7.6 (16, 191)	8.0 (46, 415)	5.3 (25, 435)	6.5 (79, 907)
DSS	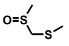	0.25	10.8 (14, 270)	9.2 (57, 560)	6.9 (23, 261)	7.0 (76, 644)
THI	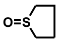	0.2	11.0 (4, 39)	10.5 (20, 132)	9.4 (20, 199)	9.1 (51, 418)

Table S-1: Robustness of TSE definition and Hammond behavior. Each column lists the average distances between the centers of mass of the ligand and FKBP active site for the conformations at the TSE. The numbers of TSE nodes and snapshots are shown in parentheses. Only TSE nodes with weight larger than 5 were used for this analysis as nodes with very low weight increase the noise [2].

8 Diffusivity test

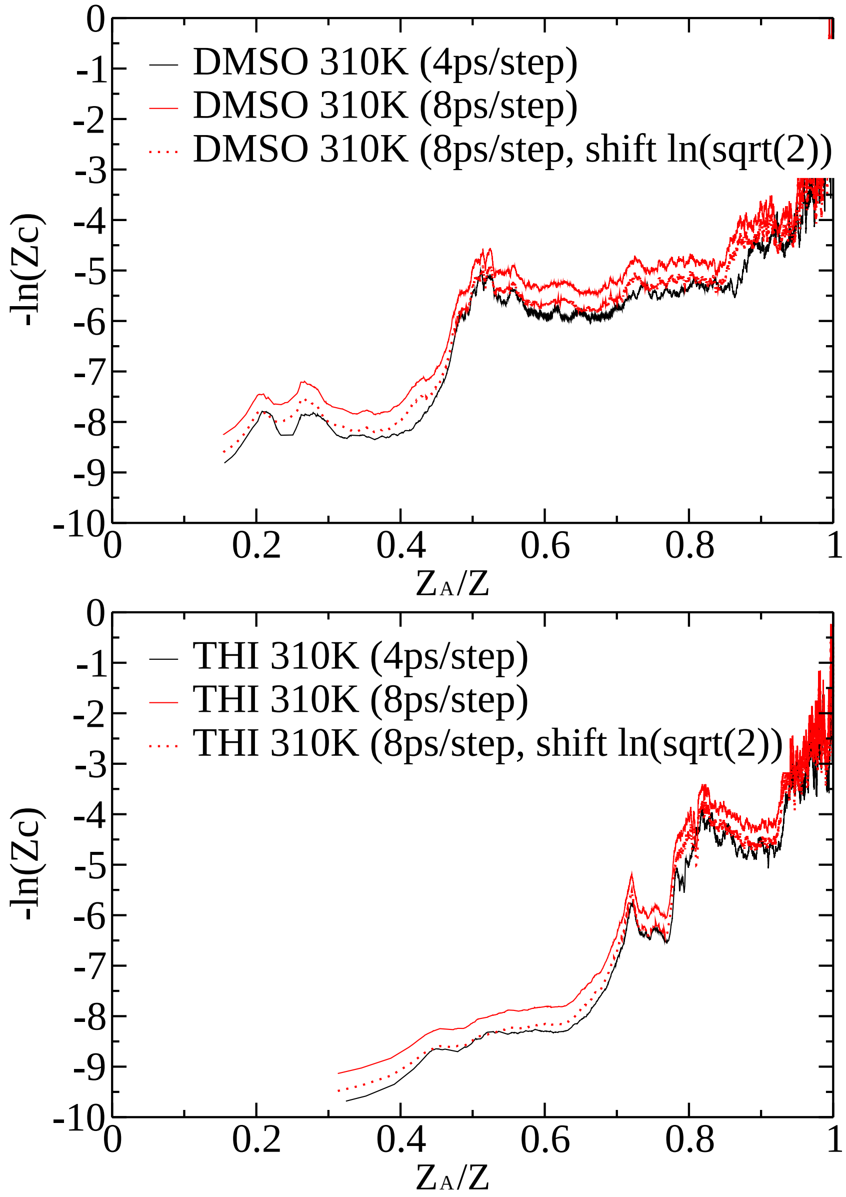

Figure S23: Diffusivity test for the clustering of DMSO and THI. The profiles with saving frequency at 4 and 8 ps are similar upon a vertical shift of $\ln(\sqrt{2})$, which is consistent with the diffusive regime [1].

9 References

References

[1] Krivov SV, Karplus M (2008) Diffusive reaction dynamics on invariant free energy profiles. Proc Natl Acad Sci USA 105:13841–13846.

[2] Muff S, Caflisch A (2009) Identification of the protein folding transition state from molecular dynamics trajectories. J Chem Phys 130:125104.
